# Predictive modeling targets thymidylate synthase ThyX in *Mycobacterium tuberculosis*

**DOI:** 10.1038/srep27792

**Published:** 2016-06-10

**Authors:** Kamel Djaout, Vinayak Singh, Yap Boum, Victoria Katawera, Hubert F. Becker, Natassja G. Bush, Stephen J. Hearnshaw, Jennifer E. Pritchard, Pauline Bourbon, Peter B. Madrid, Anthony Maxwell, Valerie Mizrahi, Hannu Myllykallio, Sean Ekins

**Affiliations:** 1 LOB, Ecole polytechnique, CNRS, INSERM, Université Paris-Saclay, 91128 Palaiseau cedex, France; 2 MRC/NHLS/UCT Molecular Mycobacteriology Research Unit & DST/NRF Centre of Excellence for Biomedical TB Research, Institute of Infectious Diseases and Molecular Medicine and Division of Medical Microbiology, Faculty of Health Sciences, University of Cape Town, AnzioRoad, Observatory 7925, Rondebosch, Cape Town 7700, South Africa; 3 Epicentre Mbarara Research Centre, Mbarara, Uganda; 4 Microbiology Department, Faculty of Medicine, Mbarara University of Science and Technology, Mbarara, Uganda; 5 Sorbonne Universités, UPMC Univ Paris 06, 4 Place Jussieu, 75005 Paris France; 6 Department of Biological Chemistry, John Innes Centre, Norwich Research Park, Norwich NR4 7UH, UK; 7 SRI International, 333 Ravenswood Avenue, Menlo Park, CA 94025, USA; 8 Collaborative Drug Discovery, 1633 Bayshore Highway, Suite 342, Burlingame, CA 94403, USA; 9 Collaborations in Chemistry, 5616 Hilltop Needmore Road, Fuquay-Varina, NC 27526, USA

## Abstract

There is an urgent need to identify new treatments for tuberculosis (TB), a major infectious disease caused by *Mycobacterium tuberculosis* (*Mtb*), which results in 1.5 million deaths each year. We have targeted two essential enzymes in this organism that are promising for antibacterial therapy and reported to be inhibited by naphthoquinones. ThyX is an essential thymidylate synthase that is mechanistically and structurally unrelated to the human enzyme. DNA gyrase is a DNA topoisomerase present in bacteria and plants but not animals. The current study set out to understand the structure-activity relationships of these targets in *Mtb* using a combination of cheminformatics and *in vitro* screening. Here, we report the identification of new *Mtb* ThyX inhibitors, 2-chloro-3-(4-methanesulfonylpiperazin-1-yl)-1,4-dihydronaphthalene-1,4-dione) and idebenone, which show modest whole-cell activity and appear to act, at least in part, by targeting ThyX in *Mtb*.

Tuberculosis (TB) is a major infectious disease that knows no geographic boundary and accounts for 9 million new cases and approximately 1.5 million deaths each year^1^. TB and its etiological agent *Mycobacterium tuberculosis* (*Mtb*) are the focus of intense efforts to develop new tools for the control and eventual elimination^2^ of this devastating disease, which is associated increasingly with resistance to first- and second-line drugs^3^. The discovery of new TB drug candidates with novel mechanisms of action that can overcome resistance, shorten the duration of treatment, and be co-administered with antiretrovirals, is of fundamental importance in this regard^4^,^5^,^6^. Over the last decade, there has been considerable investment in TB drug discovery with particular emphasis on the use of high-throughput phenotypic screening of libraries of thousands to hundreds of thousands of molecules for “hit” identification^5^,^7^,^8^,^9^. Whole-cell approaches have the advantage of allowing the high-throughput screening (HTS) assay to be conducted under conditions that mimic host infection without knowledge of mechanism of action^10^. Importantly, all presently used antibiotics have been developed by this approach, including the recently approved drug, bedaquiline^11^. Moreover, the phenotypic screening format produces a wealth of data that can be used for computational machine learning^12^, which has the potential to improve the screening efficiency of additional compounds^13^,^14^ and assist lead optimization^15^.

An alternative approach to hit identification is target-based, which relies on the availability of purified protein against which a HTS can be performed^10^ and/or knowledge of the target, such as the crystal structure, to guide structure-based drug design. Drawbacks of this approach include the difficulty in balancing target activity with the physicochemical properties needed to enter whole cells and evade efflux. This approach also requires extensive validation of the target. A recent review summarized the results of target-based and phenotypic screens conducted at the Novartis Institute for Tropical Diseases. After failing with target-based screens, phenotypic screens led to the identification of 5 chemical series in 7 years. Of these, 3 series were terminated due to glycerol-dependent activity which is irrelevant in *Mtb*, lack of *in vivo* activity, or limited maximum exposure^10^. Target-based screening has also been reviewed to identify the key properties of promising targets such as essentiality for growth, vulnerability, druggability, reduced propensity for resistance, and target localization as well as amenability to chemotherapy^16^.

For a known target, computational approaches such as docking the molecules (into the protein structure), quantitative structure-activity relationship (QSAR), pharmacophore or machine learning models can be developed to screen chemical libraries^12^. We have previously used 3D pharmacophore models, alone or in combination with Bayesian models to identify compounds with antitubercular whole-cell activity^17^,^18^, as a bridge between phenotypic screening and rational structure-based drug design.

The current study focuses on naphthoquinone (NQ) compounds which have widely reported biological activities including anti-cancer and anti-malarial activities. For instance, atovaquone (2-(trans-4-(P-chlorophenyl)cyclohexyl)-3-hydroxy-1,4-naphthoquinone), a well-known 2-OH-1,4-NQ, targets the respiratory electron transfer chain, and is clinically used in anti-pneumocystis, anti-toxoplasmosis and anti-malarial treatments. NQs also have anti-microbial activity against different bacterial pathogens, including *Mtb*^19^,^20^,^21^,^22^,^23^,^24^,^25^. Recently, we showed that NQ-based compounds inhibit the activities of PBCV-1 and *Helicobacter pylori* thymidylate synthase ThyX^26^,^27^ as well as *Mtb* DNA gyrase^28^. These observations led us to investigate inhibition of *Mtb* ThyX by NQs and develop pharmacophore models for these two essential enzymes that are both required for DNA replication^29^.

ThyX is an essential thymidylate synthase (TS) that is both mechanistically and structurally unrelated to the analogous human enzyme^30^,^31^. These enzymes catalyze the methylation of 2′-deoxyuridine-5′-monophosphate (dUMP) to synthesize 2′-deoxythymidine-5′-monophosphate (dTMP), an essential DNA precursor. In this reaction, 5,10-methylenetetrahydrofolate (CH_2_H_4_folate) and nicotinamide adenine dinucleotide phosphate (NADPH) are used as carbon and hydride donors, respectively. In the case of *Paramecium bursaria chlorella virus-1* ThyX, structural data have revealed stacking of NQ against the flavin adenine dinucleotide (FAD) co-factor, partially overlapping with the dUMP-binding pocket^27^. As dUMP acts in the ThyX reaction both as the activator and the substrate^32^, NQ binding at the ThyX active site results in potent inhibition of ThyX activity. Importantly, unlike human TS, ThyX produces tetrahydrofolate (H_4_folate) as a byproduct explaining why many *thyX*-carrying organisms do not require dihydrofolate reductase (FolA)^33^. Strikingly, although mycobacteria possess two distinct families of thymidylate synthases, the canonical ThyA as well as the non-canonical ThyX, only ThyX is essential in these organisms^34^. ThyX thus represents a promising target against *Mtb*^34^,^35^, with a crystal structure available [PDB:2AF6^36^]. Several laboratories have developed dUMP analogs to target *Mtb* ThyX, although most of the hits to date are non-selective and also inhibit ThyA^37^,^38^. More recently, conditional depletion of ThyX was shown to result in modest hypersensitivity of *Mtb* to the thymidylate synthase inhibitor and anticancer drug, 5-fluorouracil (5-FU)^39^, suggesting that inhibition of ThyX through metabolic conversion of 5-FU to 5-FdUMP comprises one element of the complex mechanism of anti-tubercular action of this drug.

NQs have also been shown to be active against DNA gyrase^28^ and appear to bind at the N-terminal domain of GyrB^26^ at a novel site that is distinct from the ATPase active site and the well-established binding site for aminocoumarin antibiotics^40^. This enzyme is a topoisomerase present in bacteria and plants but not animals, and is a validated target for antibacterials that include the fluoroquinolones, which are important second-line drugs for TB. It consists of two subunits, GyrA and GyrB, which form an A_2_B_2_ complex in the active enzyme. DNA gyrase catalyzes supercoiling of DNA in an ATP-dependent reaction; the ATPase site resides in the GyrB subunit^41^.

The observed overlap of NQs binding and inhibiting both ThyX and GyrB from *Mtb* motivated the current study to identify new inhibitors suggested using computational approaches.

## Results

### Identification of NQs as inhibitors of ThyX and gyrase

**Figure 1:**
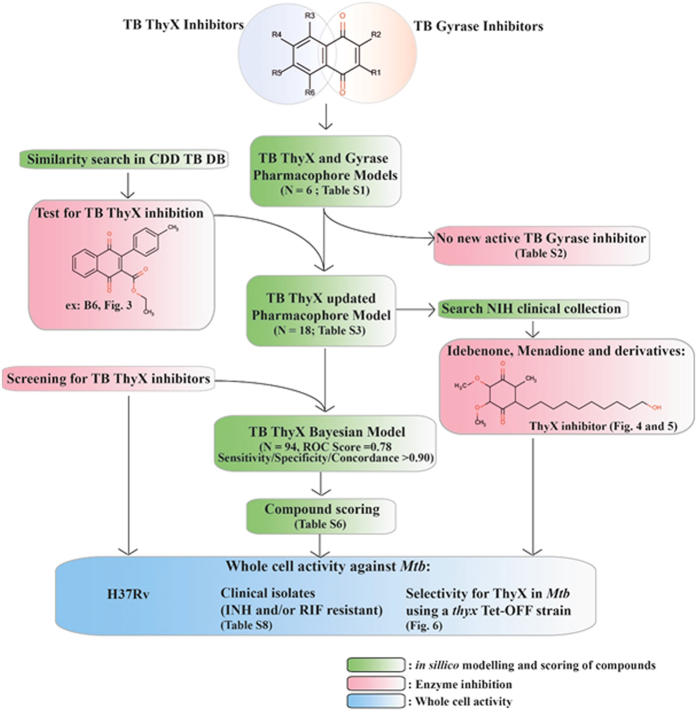
Workflow for combined computational and experimental approaches. *In silico* modelling and scoring of compounds is boxed in green. Enzyme assays are boxed in pink. Whole cell activity measurements are boxed in blue.

In this study, we utilized a combined computational and experimental workflow (Fig. 1) to obtain new insight into *Mtb* ThyX and DNA gyrase inhibition, and identify new inhibitors in the case of ThyX. A starting point for the study was the identification of NQs as inhibitors of *Mtb* ThyX and DNA gyrase (Supplementary Table 1). The compounds 2EO4 and C8-C1, originally identified as the inhibitors of the *PBCV-1* ThyX enzyme, were found to also inhibit *Mtb* ThyX, but were inactive against *Mtb* gyrase. Diospyrin inhibits only *Mtb* gyrase whereas other tested molecules showed comparable activity against both enzymes (Supplementary Table 1). These results revealed that selective or dual inhibition of these enzymes is feasible and prompted further computational analyses to identify additional inhibitors.

### Substructure searching and common features pharmacophores used for virtual screening with ThyX

**Figure 2:**
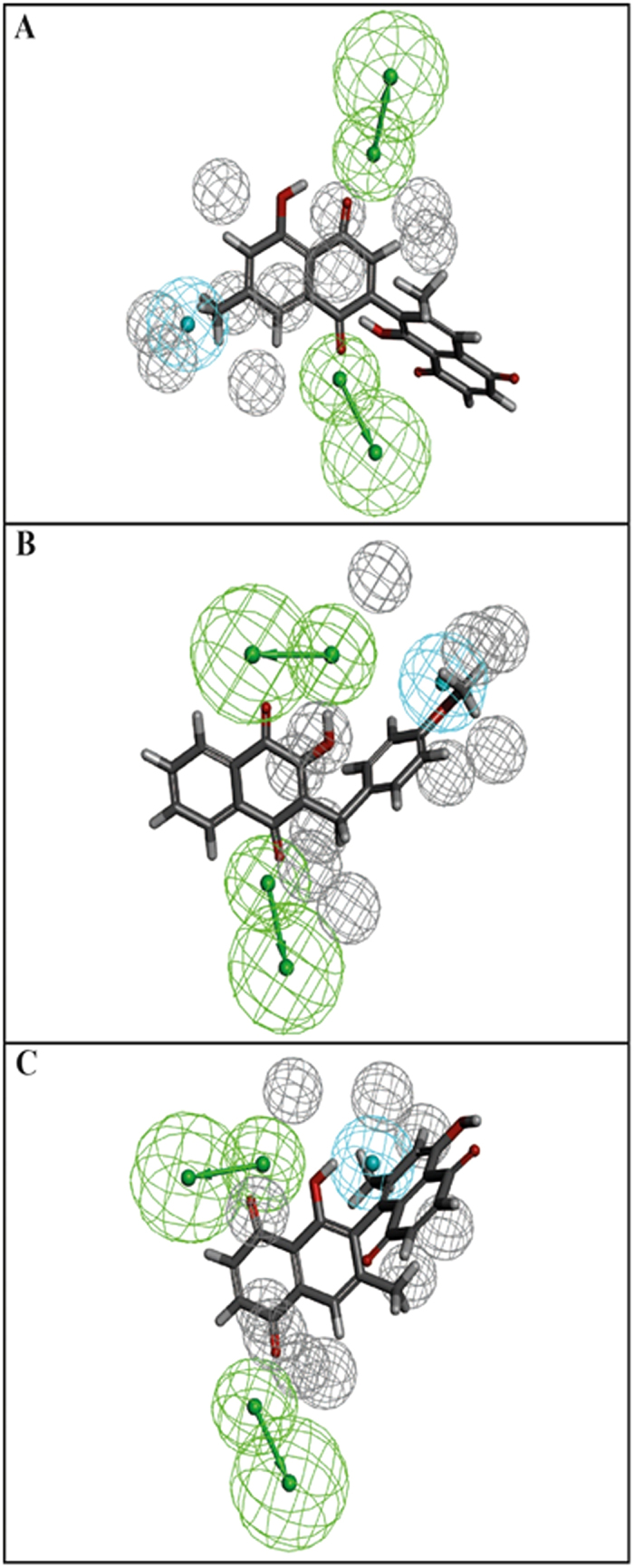
Initial common feature pharmacophores for ThyX and GyrB. (**A**) Diospyrin mapped to the GyrB model, (**B**) C8-C1 mapped to ThyX model. (**C**) ThyX model was used to score isodiospyrin (the fit was poor 0.004). Blue = hydrophobe, green = hydrogen bond acceptor, grey = excluded volume.

**Figure 3:**
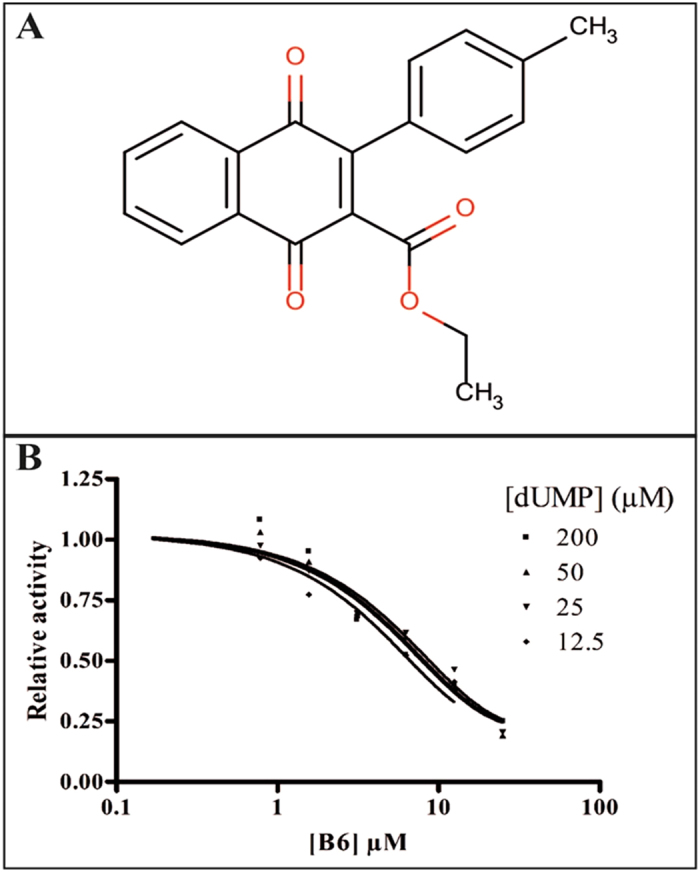
Structure and dose response curve for B6 against ThyX. (**A**) Structure of ethyl 3-(4-methylphenyl)-1,4-dioxonaphtalene-2-carboxylate (B6), a NQ identified by similarity searching in the CDD Public database of previously tested compounds against *Mtb* with known whole cell activity. (**B**) B6 dose response curves versus ThyX.

**Figure 4:**
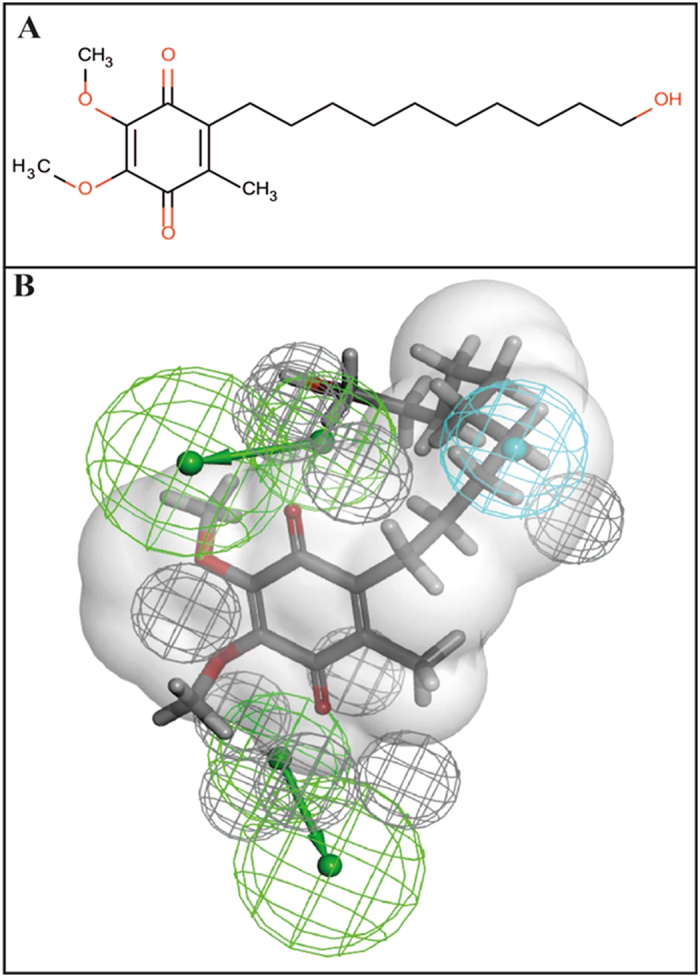
Idebenone mapped to the ThyX N = 18 pharmacophore model. (**A**) idebenone 2D structure. (**B**) Pharmacophore showing van der Waals surface based on C8-C1, Blue = hydrophobe, green = hydrogen bond acceptor, grey = excluded volume.

**Figure 5:**
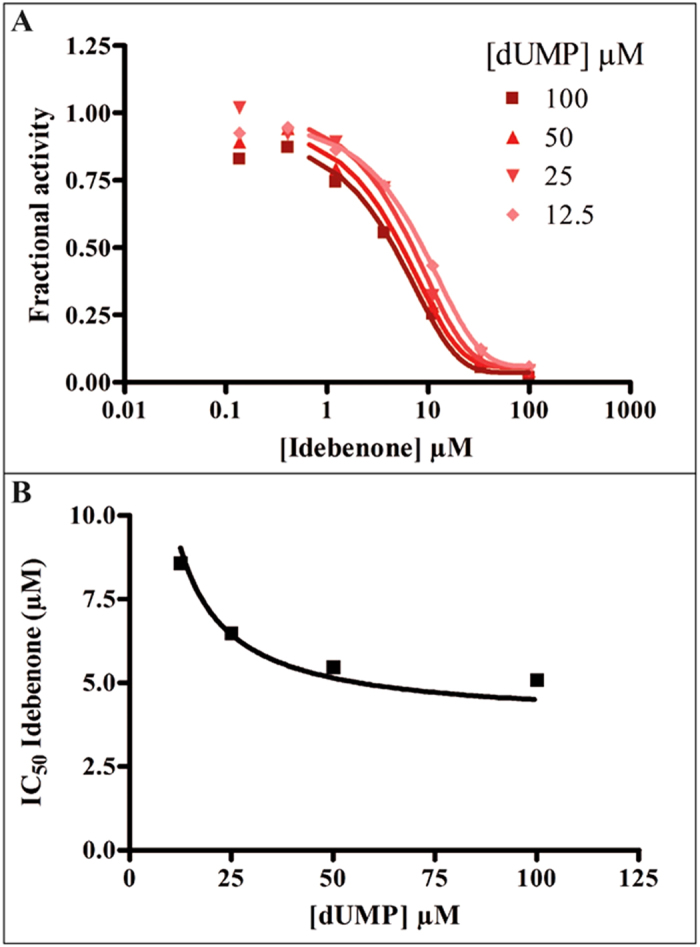
Idebenone dose response against ThyX and effect of dUMP. (**A**) Idebenone dose response curves versus ThyX. (**B**) Effect of the dUMP concentration on the IC_50_ value of Idebenone.

Using the experimental data described in Supplementary Table 1, we were able to build common features pharmacophores for *Mtb* ThyX and gyrase that consisted of excluded volumes, two hydrogen bond acceptors and one hydrophobic feature (Fig. 2). The *Mtb* GyrB pharmacophore used 6 NQs (Fig. 2A) and resulted in the same features as for the *Mtb* ThyX pharmacophore (Fig. 2B), albeit in a different arrangement. Isodiospyrin which inhibits GyrB was predicted to have a poor fit score against *Mtb* ThyX, as shown in Fig. 2C. After similarity searching previously identified whole-cell active compounds in the CDD TBDB^42^,^43^, using the napthoquinone substructure we identified a *Mtb* ThyX inhibitor, ethyl 3-(4-methylphenyl)-1,4-dioxonaphtalene-2-carboxylate (molecule B6, Fig. 3A), with a K_i_ of 4.5 μM (Fig. 3B). This molecule as well as others screened in this process were added into the models to update them. All 19 compounds that we selected for GyrB at this stage were inactive (Supplementary Table 2); however, our approach led to additional compounds that showed substantial activity against ThyX. For example, one pharmacophore with 18 molecules (N18 Pharmacophore, Supplementary Table 3) was used to search the NIH clinical collection of over 700 compounds, and one quinone compound, idebenone (Fig. 4A), was selected for testing based on its fit to the model (Fig. 4B). This molecule was found to be an uncompetitive inhibitor of *Mtb* ThyX with respect to dUMP (K_i_ = 3.3 μM, Fig. 5), suggesting that it binds preferentially to the ThyX-dUMP complex. It also has weak whole-cell activity against *Mtb*, (MIC_90_ = 125 μM; Supplementary Table 4). This table also shows the whole-cell activity of several other compounds against replicating *Mtb*, including B6, D4, D5, E1, E10, F1 and F2 which were identified by screening against the *Mtb* ThyX (Fig. 1). Interestingly, menadione inhibited growth of *Mtb* while some additional analogs were less active or had solubility issues (Supplementary Table 5).

### Predicting new ThyX inhibitors using Bayesian machine learning models

In prior work, we have used Bayesian machine learning to build models of whole-cell screens of small molecule compounds active against *Mtb*^14^, which led to the identification of new active compounds^13^,^14^,^44^. After testing 94 molecules against *Mtb* ThyX at 100 μM (the training set, Supplemental Table 6), we generated a Bayesian model using molecules with >70% inhibition as actives. This resulted in a promising model with a ROC score of 0.78 after 5-fold cross validation, alongside sensitivity, specificity and concordance values greater than 0.90 (Supplementary Fig. 1A). The ‘good’ features were predominantly quinones and NQs (Supplementary Fig. 1B), while ‘bad’ features included amines and sulfonamides (Supplementary Fig. 1C). A model with similar statistics was generated in CDD models using FCFP6 descriptors alone, with a 3-fold cross validation ROC score of 0.80 (Supplementary Fig. 2).

### Experimental testing of Bayesian model for NQ inhibitors of ThyX

The Bayesian model generated with Discovery Studio was used to predict activity of 14 compounds (007B-010K, Supplementary Table 7) which were not included in the training set. The closest distance calculation uses the calculated Euclidean distance between each molecule and those in the training set and suggests they are different (a zero distance corresponds to identical molecules). Two of these molecules were predicted as “inactive” (010-I and 010-C, Supplementary Table 7). However, six of the remaining 12 compounds (50%) were predicted as actives and exhibited over 70% inhibition (the selected cut-off) of *Mtb* ThyX activity *in vitro* at 100 μM. These experimental results illustrate the robustness of the models.

### Whole-cell activity against *Mtb*

We next investigated whole-cell activity of the NQs against laboratory and clinical isolates of *Mtb* with different drug resistance profiles to standard anti-tuberculars (Supplementary Table 8) under replicating conditions. MIC_90_ values of the molecules in the test set (Supplementary Table 7) ranged from 20 to 200 μM (Supplementary Table 9).

**Figure 6:**
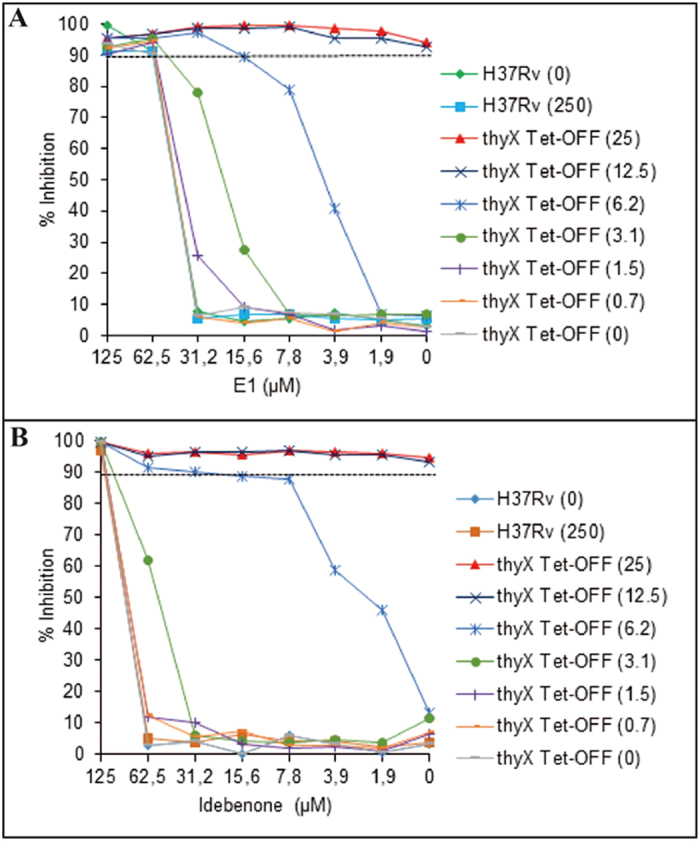
Depletion of ThyX in *Mtb* confers modest hypersensitivity to E1 and idebenone. The effect of ThyX depletion on the susceptibility to E1 and idebenone was assessed using the *thyX* Tet-OFF mutant in a checkerboard assay. The H37Rv strain was used as a control. Bacterial viability was assessed by the Alamar Blue assay, measuring fluorescence at 544/590 nm. The numbers in parentheses denote the concentration of anhydrotetracycline (ATc, ng/ml), which modulates the expression of *thyX* in *thyX* Tet-OFF^39^. Ninety percent growth inhibition (MIC_90_) is represented by the dashed horizontal line. The results shown are representative of one of the three biological replicates.

Additional compounds that either acted as MtbThyX inhibitors *in vitro* or were selected by the N18 pharmacophore model were also evaluated to identify those with growth inhibitory activity against replicating *Mtb* H37Rv (Supplementary Table 4). Molecules with whole-cell activity were then tested against a conditional knockdown mutant of *Mtb* H37Rv, *thyX* Tet-OFF, in which *thyX* is expressed under the control of a Tet-regulated promoter^39^. In this target-based whole-cell assay, compounds with ThyX-selective activity in *Mtb* can be identified on the basis of whether ThyX depletion confers hypersensitivity to the compound^39^,^45^. As expected, the positive control, 5-FU, showed a progressive, ATc-dependent shift in MIC_90_ from 3.1 μM in the absence of ATc to 0.3 μM at an ATc concentration of 6.2 ng/ml. Among the other compounds tested in this assay, two showed ≥ 4-fold increase in potency upon ThyX depletion, namely, E1 (2-chloro-3-(4-methanesulfonylpiperazin-1-yl)-1,4-dihydronaphthalene-1,4-dione)) and idebenone (Fig. 6). These compounds demonstrated the same activity against *thyX* Tet-OFF in the absence of ATc compared to the H37Rv control, with MIC_90_ values of 62.5 and 125 μM, respectively. Addition of ATc resulted in a progressive increase in susceptibility to E1, with the MIC_90_ value decreasing to 15.6 μM at an ATc concentration of 6.2 ng/ml. ThyX depletion also sensitized *Mtb* to idebenone with the MIC_90_ shifting ~5-fold, from 125 μM to 22 μM. However, for reasons that are unclear, idebenone displayed atypical behavior in this checkerboard assay compared to 5-FU^39^ and E1, showing ~90% growth inhibition over an unusually large concentration range (62.5–7.8 μM) when added to *thyX* Tet-OFF in the presence of ATc at 6.2 ng/ml (Fig. 6B). Of the other compounds tested, F2 showed a slight (~2-fold) MIC_90_ shift (62.5 to 31.2 μM) in the presence of ATc at 6.2 ng/ml, whereas no shift in MIC_90_ was observed for B6, D4, D5, E10 and F1. Together, these results implicate ThyX as a potential target for E1 and idebenone in *Mtb*.

## Discussion

We have used a combination of cheminformatics and experimental strategies (Fig. 1) to identify new inhibitors of *Mtb* ThyX. Many previous studies have shown that NQs possess activity against *Mtb*^19^,^20^,^21^,^22^,^23^,^24^,^25^,^28^ and other bacteria^27^. It has long been suggested that such quinones are reactive and generally non-specific^46^, yet these natural products are already in therapeutic use, demonstrating that selective toxicity can be attained^47^,^48^. Starting with a set of NQs active against *Mtb* ThyX and/or GyrB (Supplementary Table 1), and employing a common feature pharmacophore approach, we showed that while a small set of compounds had identical pharmacophore features in each model, their arrangement was unique (Fig. 2). As we updated these models we noted little apparent change in the *Mtb* ThyX model and retrieved several compounds of interest for testing. However, the *Mtb* GyrB pharmacophore was unable to retrieve any additional active molecules containing NQ or other features (Supplementary Table 2). One of the compounds identified as active against *Mtb* ThyX using a pharmacophore based on NQs was idebenone (Figs 4 and 5), which demonstrate that the models can also retrieve non-NQs as ThyX inhibitors. Unlike the competitive mode of inhibition of NQs against PBCV-1 ThyX, idebenone exhibited uncompetitive inhibition of *Mtb* ThyX with respect to dUMP binding suggesting that it binds to, in addition to the free enzyme, to the *Mtb* ThyX-dUMP complex^27^. Idebenone has recently completed a phase III clinical trial for Duchene Muscular Dystrophy^49^. It is a potent antioxidant^50^ and can donate electrons to complex III of the electron transport chain. This drug has also found utility in studies on mitochondrial diseases including Friedreich’s Ataxia^51^ and is approved in Europe for Leber’s Hereditary Optic Neuropathy^52^. It is therefore possible that idebenone, while exhibiting only weak activity on *Mtb* whole cells, could have potential for repurposing against additional diseases as well. We also tested menadione and analogs that had improved whole cell activity (Supplementary Table 5) compared to idebenone. Menadione was inactive against *Mtb* gyrase but 7-methyljuglone, an analogue of menadione, was active against both *Mtb* ThyX and gyrase, indicating that modification of a single hydroxyl in methyljuglone to a methyl group in menadione, participates in dual inhibition of *Mtb* ThyX and gyrase (Supplementary Table 1).

As we collected and tested a relatively large number of molecules (N = 94) against *Mtb* ThyX, we were able to use a machine learning approach. To our knowledge this represents the first target-based machine learning approach towards the identification of enzyme inhibitors in *Mtb*. Clearly, quinone and NQ substructures were identified as important for activity and molecules with amines and sulfonamides were less desirable. These machine learning results could help us focus on compounds to test in the future. The Bayesian model has the added advantage of being able to quickly score compounds so could narrow down potential compounds for testing. We evaluated the Bayesian model with additional NQs (Supplementary Table 7). Our model predicted potent new *Mtb* ThyX inhibitors that had some whole-cell activity against *Mtb*, thus attesting to the utility of our approach. In addition we have used a second software tool (CDD Models) which produces a similar leave-out-cross-validation ROC value, with the advantage that this Bayesian model can be shared openly with others so that they can benefit from these modeling efforts^53^.

In this study we had greater success identifying additional inhibitors of *Mtb* ThyX than GyrB. While there is some inhibition overlap revealed by the NQs, this could suggest that the chemical property/feature permissiveness of *Mtb* ThyX is greater than GyrB due to differences in the binding site interactions. Substructure searching for compounds previously identified in whole-cell screens against *Mtb* identified a NQ (B6) with activity against *Mtb* ThyX (Fig. 3) and moderate antibacterial activity on *Mtb* (Supplementary Table 4). Idebenone, while not particularly active in whole *Mtb* cells may target ThyX as part of its mechanism of action in *Mtb*. Therefore, idebenone could be a starting point for structure-based design or by creating new derivatives using our previous Bayesian models based on whole cell activity data^14^. The progress of idebenone in clinical trials and as an approved drug suggests that it is likely very acceptable from the point of view of physicochemical properties, formulation and safety.

In future work, new compounds could be evaluated with our previously published Bayesian models for whole cell activity^14^ alongside the current *Mtb* ThyX Bayesian model in order to prioritize molecules for testing. This would enable the optimization of both target and whole cell activity in parallel. We propose that a combination of pharmacophore modeling, target-based whole-cell assay and resultant machine learning molecules using this data could result in the identification of novel scaffolds for pursuit.

## Methods

### Compounds

Tested compounds were purchased from ChemBridge, Vitas-M Laboratory Ltd., InterBioScreen or Sigma and were >90% pure (see Supplementary Table 6 for more details).

### ThyX assays

*Mtb* ThyX activity was measured using the tritium release assay, as previously described for *Helicobacter pylori* ThyX^26^. Reaction mixture included 10 mM MgCl_2_, 300 mM NaCl, 500 μM FAD, bovine serum albumin (200 μg/ml), 250 μM CH_2_H_4_folate (Eprova, Merck), 1 mM NADPH, 100 μM dUMP, [5H^3^]dUMP (Moravek Biochemicals, CA, USA) and 1 μM *Mtb* ThyX in 50 mM HEPES pH 8. DMSO concentration was maintained constant at 1%. Reactions were initiated by addition of NADPH (1 mM) at 37 °C and were stopped after 7 mins. For idebenone, IC_50_ value decreased as a function of increasing dUMP concentration indicating an un-competitive mode of inhibition. Therefore, the formula IC_50_ = *Ki* * (1 + *K*_*m*_/[*S*]) + [*E*]/2 where *K*_*m*_ is the Michaelis constant for dUMP, [S] is the dUMP concentration, and [E] is the enzyme concentration in the assay, was used to convert a measured IC_50_ value to the corresponding *K*_*i*_.

### Gyrase assays

*Mtb* gyrase supercoiling assays were carried out as described previously^54^.

### Drug susceptibility testing against *Mtb*

A resazurin (Alamar Blue) assay was used to assess activity against strains of *Mtb*^55^. The antimicrobial susceptibility test was performed in a clear-bottomed, round well, 96-well microtiter plate. Compounds were tested at 8 concentrations ranging between 40 and 0.31 μg/mL with a final DMSO concentration of 1.25% in each well. After a growth medium containing ~10^4^ bacteria was added to each well, the different dilutions of compounds were added. Controls included wells containing concentrations of rifampin and isoniazid ranging from 0.00039 to 8.0 μg/mL to control for assay performance; wells with bacteria, growth medium, and vehicle (1.25% DMSO); and sterility control wells with medium. Plates were incubated at 37 °C for 6 d in an ambient incubator at which time 5 μL of 1% resazurin dye was added to each well. After 2 days of incubation, visual inspection of color (pink, periwinkle or blue) was recorded for each well along with measurements of fluorescence in a microtiter plate fluorimeter with excitation at 530 nm and emission at 590 nm. The lowest drug concentration that inhibited growth of ≥90% of *Mtb* bacilli in the broth was considered the MIC_90_ value^56^. Rifampicin and isoniazid were used as positive controls and were consistently in the acceptable range. Values were converted from μg/ml to μM throughout the paper, for consistency.

### Target-based whole-cell screening

The susceptibility of *Mtb* H37Rv and a conditional knockdown of ThyX in *Mtb*, *thyX* Tet-OFF, to a subset of test compounds was also determined by the broth microdilution method, as described previously^39^. Briefly, bacteria were grown in Middlebrook 7H9 broth (BD) supplemented with OADC (BD), 0.2% glycerol and 0.05% Tween-80 to mid-exponential phase. The culture was diluted and ~10^5^ CFU/ml was added to a 96-well microtiter plate containing 2-fold serial dilutions of drug which was then incubated at 37 °C. For the pairwise combination (anhydrotetracycline (ATc) vs. test compound) assay using the *thyX* Tet-OFF strain, a two-dimensional array of serial dilutions of ATc and the test compound was prepared in a 96-well plate. Control wells consisting of bacteria only or medium only were treated with the same concentration of DMSO as used in drug-containing wells. At day 7, 10 μl of Alamar Blue solution (Invitrogen) was added and plates were reincubated at 37 °C. After 24 h, fluorescence (excitation 544 nm; emission 590 nm) was measured in a FLUOstar OPTIMA plate reader (BMG LABTECH, Offenberg, Germany). Data were normalised to the minimum and maximum inhibition controls to generate a dose response curve (% inhibition) from which the MIC_90_ was determined.

### Substructure searching

The CDD database (Collaborative Drug Discovery Inc. Burlingame, CA) has been described previously and applied for TB research^14^. The literature data on *Mtb* drug discovery has been curated and 26 *Mtb* specific datasets (including published data from high throughput screens performed by NIAID/Southern Research Institute have been compiled in the CDD Public database) are hosted, representing over 350,000 compounds derived from patents, literature and HTS data, and we have termed this the CDD TB DB. The NQ substructure was used to query these NIAID/Southern Research Institute data. Molecules which showed previously published good whole-cell activity (MIC_90_) were selected for testing. The data generated in this study were collected and uploaded in the CDD private Vault (http://www.collaborativedrug.com/register) from sdf files and mapped to custom protocols.

### Common features pharmacophores

A set of NQs (Supplemental Table 1) was used to build common features pharmacophores for *Mtb* ThyX and GyrB with Discovery Studio 4.1 (Biovia, San Diego, CA) from 3D conformations of the molecules generated with the CAESAR algorithm. This identified key features for each protein. The pharmacophores were then used to search various databases (for which up to 100 molecule conformations with the FAST conformer generation method with the maximum energy threshold of 20 kcal/mol, were created) such as the NIH clinical drugs set containing over 700 molecules. The pharmacophore models were updated as additional data were generated.

### Bayesian models

We have previously described the generation and validation of the Laplacian-corrected Bayesian classifier models for *Mtb*^14^ using Discovery Studio 3.5 (San Diego, CA). The models were all generated using the following molecular descriptors: molecular function class fingerprints of maximum diameter 6 (FCFP_6), AlogP, molecular weight, number of rotatable bonds, number of rings, number of aromatic rings, number of hydrogen bond acceptors, number of hydrogen bond donors, and molecular fractional polar surface area which were all calculated from input sdf files. This approach was applied to the ThyX data generated in this study using the cut off of 70% inhibition at 100 μM to define actives. The resulting models were also validated using leave-one-out cross-validation; 5-fold validation to generate the receiver operator curve area under the curve (ROC AUC); concordance; specificity, and selectivity, as described previously. In the current study, as well as using the datasets individually, we also combined the datasets. Bayesian models were also generated in the CDD Vault using CDD Models, as described previously^53^. The current implementation used the FCFP6 fingerprints alone, and by default 3-fold cross-validation is performed. The model can also be exported from CDD Vault for use in other open source software and mobile apps^53^.

## Additional Information

**How to cite this article**: Djaout, K. *et al*. Predictive modeling targets thymidylate synthase ThyX in *Mycobacterium tuberculosis*. *Sci. Rep.* **6**, 27792; doi: 10.1038/srep27792 (2016).

## Supplementary Material

Supplementary Information
