# Supplementary material for: Predictive modeling targets thymidylate synthase ThyX in Mycobacterium tuberculosis
Source: Sci Rep. 2016 Jun 10;6:27792. doi: 10.1038/srep27792 (PMC4901301; doi:10.1038/srep27792)
Supplement: Supplementary Information [file srep27792-s1.pdf]

Supplementary information

Predictive modeling targets thymidylate synthase ThyX in *Mycobacterium tuberculosis*

Kamel Djaout[1], Vinayak Singh[2], Yap Boum[3,4], Victoria Katawera[3], Hubert F. Becker[1,5], Natassja G. Bush[6], Stephen J. Hearnshaw[6,¶], Jennifer E. Pritchard[6§], Pauline Bourbon[7] Peter B. Madrid[7], Anthony Maxwell[6], Valerie Mizrahi[2], Hannu Myllykallio[1*] & Sean Ekins[8,9,*]

[1]LOB, Ecole polytechnique, CNRS, INSERM, Université Paris-Saclay, 91128 Palaiseau cedex, France.

[2]MRC/NHLS/UCT Molecular Mycobacteriology Research Unit & DST/NRF Centre of Excellence for Biomedical TB Research, Institute of Infectious Diseases and Molecular Medicine and Division of Medical Microbiology, Faculty of Health Sciences, University of Cape Town, AnzioRoad, Observatory 7925, Rondebosch, Cape Town 7700, South Africa.

[3]Epicentre Mbarara Research Centre, Mbarara, Uganda.

[4]Microbiology Department, Faculty of Medicine, Mbarara University of Science and Technology, Mbarara, Uganda

[5] Sorbonne Universités, UPMC Univ Paris 06, 4 Place Jussieu, 75005 Paris (France)

[6]Department of Biological Chemistry, John Innes Centre, Norwich Research Park, Norwich NR4 7UH, UK.

[7]SRI International, 333 Ravenswood Avenue, Menlo Park, CA 94025, USA.

[8]Collaborative Drug Discovery, 1633 Bayshore Highway, Suite 342, Burlingame, CA 94403, USA

[9]Collaborations in Chemistry, 5616 Hilltop Needmore Road, Fuquay-Varina, NC 27526, USA.

Present addresses:

[¶]Stephen Hearnshaw, Inspiralis Ltd., Norwich Bioincubator, Norwich Research Park, Colney Lane, Norwich NR4 7UH, UK

[§]Jennifer Pritchard, Birkbeck College, Univ. London WC1E 7HX, UK

Running Head: ThyX inhibitors for *Mtb*

Key words: Bayesian models; Cheminformatics; thymidylate synthase; ThyX; DNA gyrase; Machine learning; *Mycobacterium tuberculosis*; Pharmacophore; naphthoquinones

Supplementary Figure 1. Bayesian Model for ThyX inhibitors A. Receiver operator curve (ROC) for 5 fold cross validation demonstrating an ROC of 0.78, B Good features in the model, C. bad features in the model.

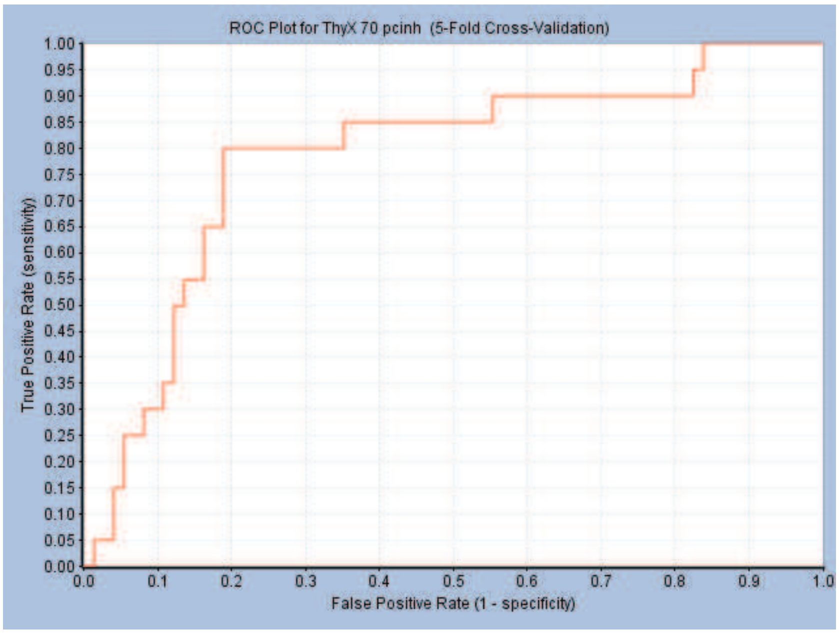

A

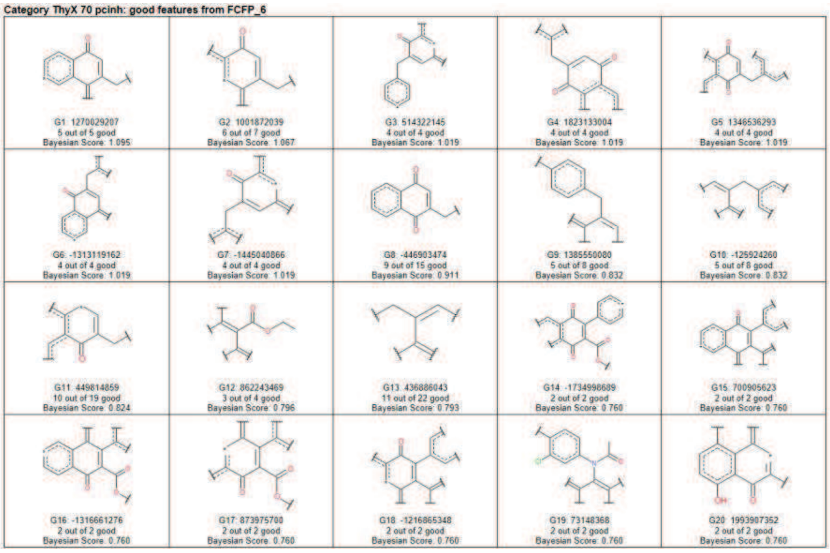

B

C

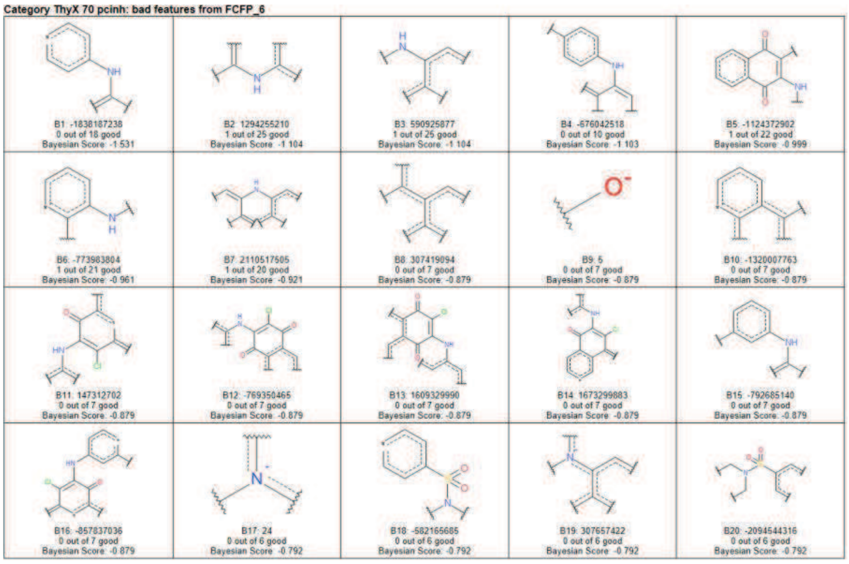
Category ThyX 70 pcinh: bad features from FCFP_6
B1: -1838187238
0 out of 18 good
Bayesian Score: -1.531
B2: 1294255210
1 out of 25 good
Bayesian Score: -1.104
B3: 590925877
1 out of 25 good
Bayesian Score: -1.104
B4: -676042518
0 out of 10 good
Bayesian Score: -1.103
B5: -1124372902
1 out of 22 good
Bayesian Score: -0.999
B6: -773983804
1 out of 21 good
Bayesian Score: -0.961
B7: 2110517505
1 out of 20 good
Bayesian Score: -0.921
B8: 307419094
0 out of 7 good
Bayesian Score: -0.879
B9: 5
0 out of 7 good
Bayesian Score: -0.879
B10: -1320007763
0 out of 7 good
Bayesian Score: -0.879
B11: 147312702
0 out of 7 good
Bayesian Score: -0.879
B12: -769350465
0 out of 7 good
Bayesian Score: -0.879
B13: 1609329990
0 out of 7 good
Bayesian Score: -0.879
B14: 1673299883
0 out of 7 good
Bayesian Score: -0.879
B15: -792685140
0 out of 7 good
Bayesian Score: -0.879
B16: -857837036
0 out of 7 good
Bayesian Score: -0.879
B17: 24
0 out of 6 good
Bayesian Score: -0.792
B18: -582165685
0 out of 6 good
Bayesian Score: -0.792
B19: 307657422
0 out of 6 good
Bayesian Score: -0.792
B20: -2094544316
0 out of 6 good
Bayesian Score: -0.792

Supplementary Figure 2. Bayesian model generated using FCFP6 descriptors only with CDD Models software (part of CDD Vault software http://www.collaborativedrug.com/register) showing 3 fold cross validation Receiver operator curve (ROC).

Protocol Definition Edit protocol definition

**Name:**	ThyX greater than 70% inh
**Category:**	QSAR Model
**Model:**	CDD's open source modified Bayesian model described in Xia et al. 2004.
**Descriptors:**	CDD's open source FCFP6 fingerprints described in Rogers and Hahn 2010.
**Description:**	

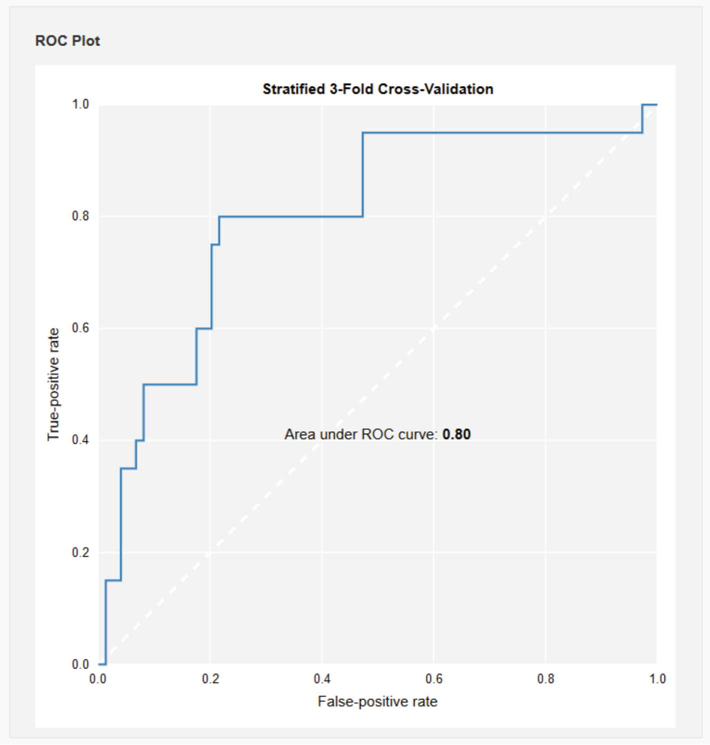

Training Set

**"Good" molecules:**	20
**Other molecules:**	74 taken from protocol TB ThyX - Hannu

Readout Definitions

Name	Data Type	Unit	Condition?	Description
**Score**	Number			Relative score (higher is better)
**Applicability**	Number			Fraction of structural features shared with the training set
**Maximum similarity**	Number			Maximum Tanimoto/Jaccard similarity to any of the "good" molecules

Supplementary Table 1. Initial molecules used for generating pharmacophores for ThyX and GyrB

Compound	Structure	IC_{50} gyrase	IC_{50} ThyX
2E04	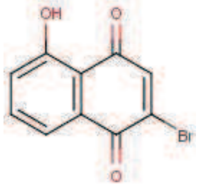	>50 μM	5 μM
C8-C1	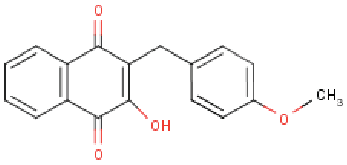	No inhibition	4.5 μM
Diospyrin	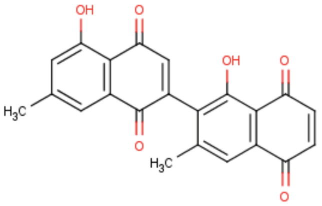	15 μM	No inhibition
Neodiospyrin	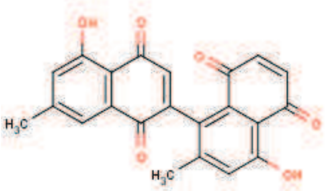	50 μM	20 μM
7-methyljuglone	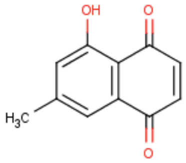	30 μM	35 μM
Isodiospyrin	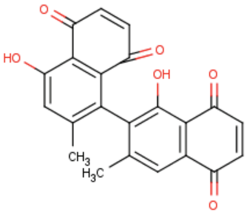	100 μM	n.d.

Supplementary Table 2. Compounds with no activity against gyrase at 100 uM

Chemical name	Chemical structure
benzyl [(4-butyl-6-chloro-2-oxo-2H-chromen-7-yl)oxy]acetate	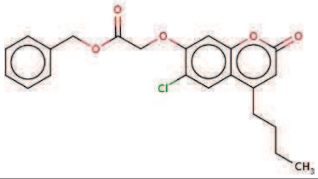
methyl {[6-ethyl-3-(4-methoxyphenyl)-4-oxo-4H-chromen-7-yl]oxy}acetate	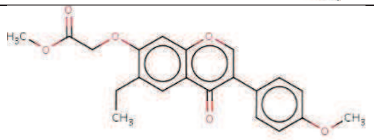
2-ethoxyethyl [(2-oxo-4-phenyl-2H-chromen-7-yl)oxy]acetate	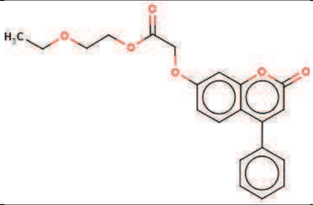
ethyl [(6-chloro-4-ethyl-2-oxo-2H-chromen-7-yl)oxy]acetate	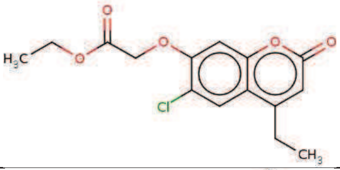
methyl [(6-chloro-2-oxo-4-phenyl-2H-chromen-7-yl)oxy]acetate	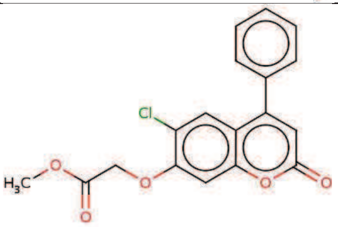
methyl [(6-chloro-2-oxo-4-propyl-2H-chromen-7-yl)oxy]acetate	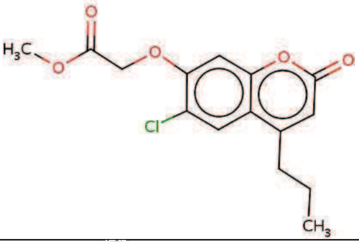
prop-2-en-1-yl 2-[(3-benzyl-6-chloro-4-methyl-2-oxo-2H-chromen-7-yl)oxy]acetate	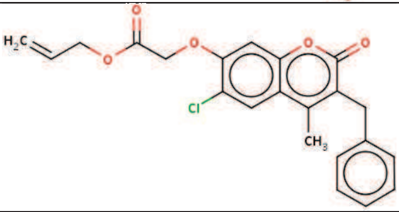

3-(3-allyl-2-imino-4-oxo-1,3-thiazolidin-5-ylidene)-1,3-dihydro-2H-indol-2-one	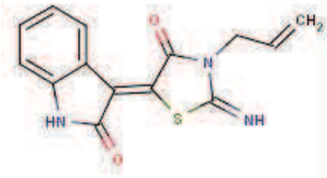
2,3-dihydro-1H-Tbenzo[b]pyrido[3,2,1-kl]phenoxazine-8,13-dione	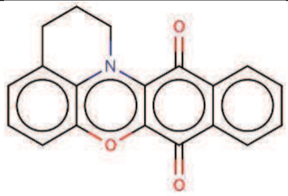
Menadione	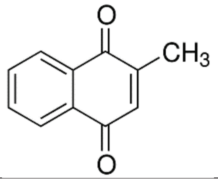
Genistein	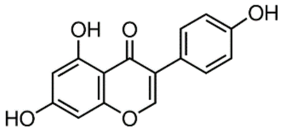
Idebenone	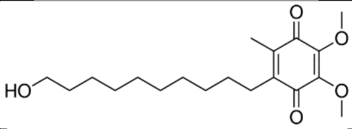
Taxifolin	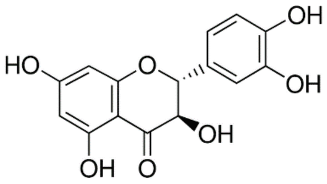
ethyl 3-(4-methylphenyl)-1,4-dioxonaphthalene-2-carboxylate (6)	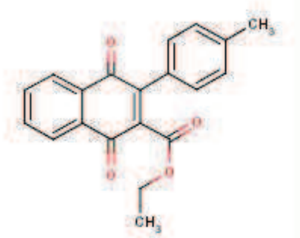
Secoisolariciresinol	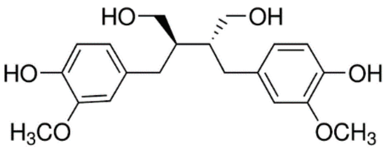
Meropenem	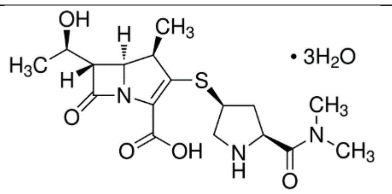

Pravastatin	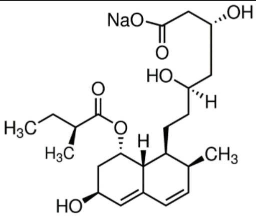
Dexamethasone	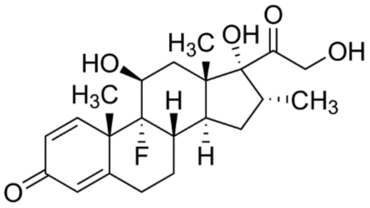

Supplementary Table 3. Molecules in ThyX pharmacophore model with 18 molecules (N18)

Molecule Smiles	Identifier	Principal	Max omit features
Oc1cccc2C(=O)C(=CC(=O)c12)Br	cpd1	2	0
COc1ccc(CC2=C(O)C(=O)c3ccccc3C2=O)cc1	cpd2	2	0
Cc1cc(O)c2C(=O)C=C(C(=O)c2c1)c3c(C)cc4C(=O)C=CC(=O)c4c3O	cpd3	0	0
Cc1cc(O)c2C(=O)C=C(C(=O)c2c1)c3c(C)cc(O)c4C(=O)C=CC(=O)c34	cpd4	1	0
Cc1cc(O)c2C(=O)C=CC(=O)c2c1	cpd5	1	0
CCN(CC)c1ccc(NC2=C(Cl)C(=O)c3ccccc3C2=O)cc1	cpd7	0	0
CCOC(=O)C1=C(C(=O)c2ccccc2C1=O)c3ccc(C)cc3	cpd8	2	0
CC(=O)c1c(C)[n+]([O-])c2ccccc2[n+]1[O-]	cpd9	0	0
[O-][n+]1c2ccccc2[n+]([O-])c3ccccc13	cpd10	0	0
CC(=O)c1c(C)[n+]([O-])c2cc(Cl)c(Cl)cc2[n+]1[O-]	cpd11	0	0
O=C1C2=C(N3CCCc4cccc(O2)c34)C(=O)c5ccccc15	cpd12	0	0
CCCCC1=CC(=O)Oc2cc(OCC(=O)OCc3ccccc3)c(Cl)cc12	6661898	0	0
CCc1ccc(cc1)C2=COc3cc(OCC(=O)OC)c(CC)cc3C2=O	6939246	0	0
CCOCCOC(=O)COc1ccc2C(=CC(=O)Oc2c1)c3ccccc3	6970845	0	0
CCOC(=O)COc1cc2OC(=O)C=C(CC)c2cc1Cl	6653343	0	0
COC(=O)COc1cc2OC(=O)C=C(c3ccccc3)c2cc1Cl	5618162	0	0
CCCC1=CC(=O)Oc2cc(OCC(=O)OC)c(Cl)cc12	5753116	0	0
CC1=C(Cc2ccccc2)C(=O)Oc3cc(OCC(=O)OCC=C)c(Cl)cc13	7105644	0	0

Supplementary Table 4. Anti-tubercular activities of compounds identified by various screening methods

Name	Structure	MIC_{90} (μM) against H37Rv
B6	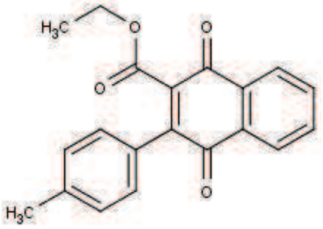	62.5
D4	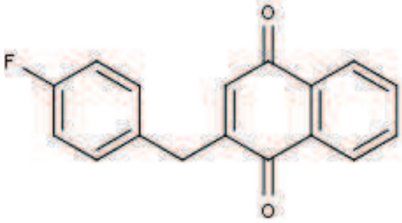	31.2
D5	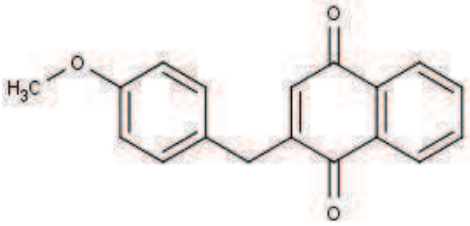	62.5
E1	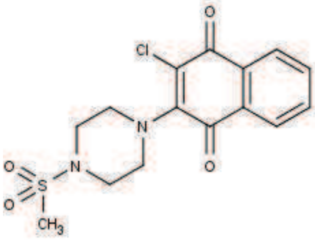	62.5
E10	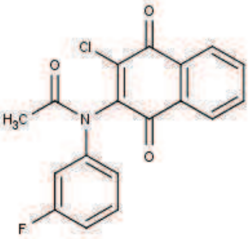	31.2

F1	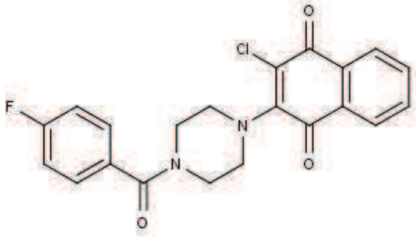	31.2
F2	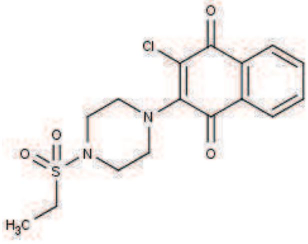	31.2
Idebenone	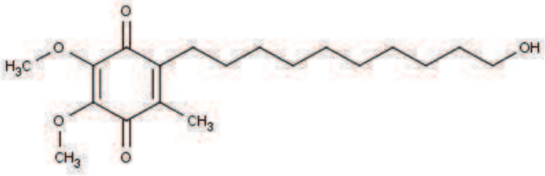	125.0

Supplementary Table 5. *Mtb* growth inhibitory activities of menadione analogs. *5 μg/ml was the highest concentration soluble in 7H9 media with 1% ethanol as a co-solvent.

Molecule	Structure	MIC (H37Rv) μM
MK-9	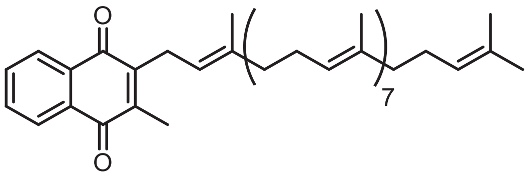	>6.4*
Menadione	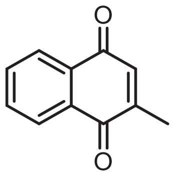	98.8
Menadione Bisulfate	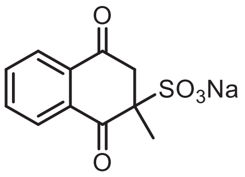	311.3

Supplementary Table 6. 94 molecules tested against Mtb ThyX at 100 μM

Structure	Smiles	Vendor source	ID	Purity	Inhibition at 100uM (%)	Activity class
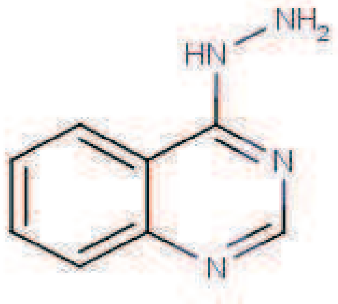	NNC1=NC=NC2=C1C=CC=C2	Chembridge (Hit2Lead)	4004613	>90%	<20	0
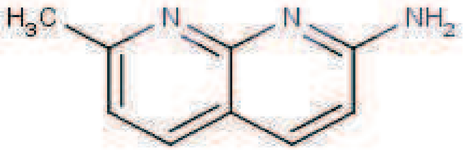	CC1=CC=C2C=CC(N)=NC2=N1	Chembridge (Hit2Lead)	4017230	>90%	<20	0
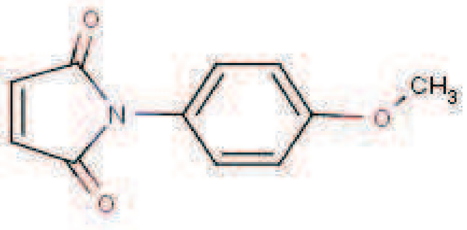	COC1=CC=C(C=C1)N1C(=O)C=CC1=O	Chembridge (Hit2Lead)	5105199	>90%	<20	0

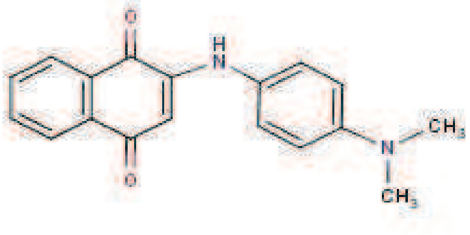	CN(C)C1=CC=C(NC2=CC(=O)C3=C(C=CC=C3)C2=O)C=C1	Chembridge (Hit2Lead)	5106780	>90%	<20	0
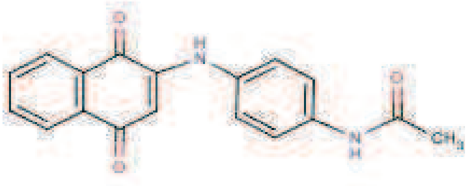	CC(=O)NC1=CC=C(NC2=CC(=O)C3=C(C=CC=C3)C2=O)C=C1	Chembridge (Hit2Lead)	5106790	>90%	20-70	0
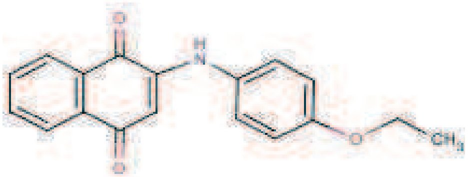	CCOC1=CC=C(NC2=CC(=O)C3=C(C=CC=C3)C2=O)C=C1	Chembridge (Hit2Lead)	5106794	>90%	<20	0

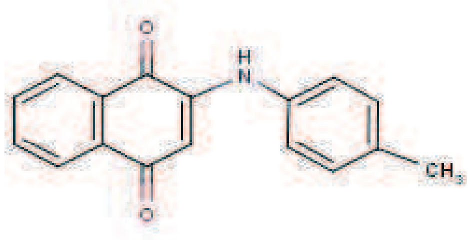		CCOC1=CC=C(NC2=CC(=O)C3=C(C=CC=C3)C2=O)C=C1	Chembridge (Hit2Lead)	5107105	>90%	<20	0
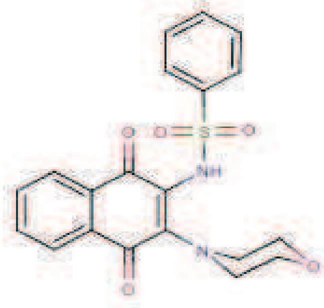		O=C1C(NS(=O)(=O)C2=CC=CC=C2)=C(N2CCOCC2)C(=O)C2=C1C=CC=C2	Chembridge (Hit2Lead)	5123492	>90%	<20	0
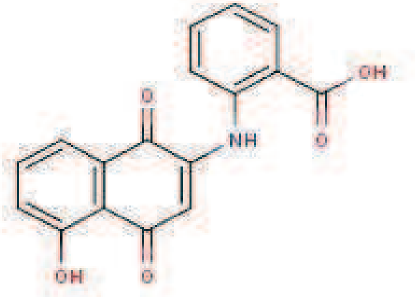		OC(=O)C1=CC=CC=C1NC1=CC(=O)C2=C(C=CC=C2O)C1=O	Chembridge (Hit2Lead)	5138303	>90%	70-90	0

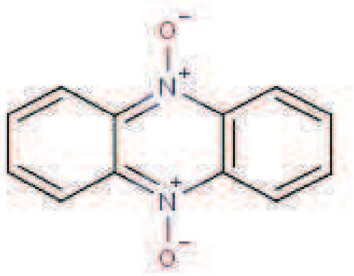 .	O[N+]1=C2C=CC=CC2=[N+](O)C2=C1C=CC=C2	Chembridge (Hit2Lead)	5141532	>90%	20-70	0
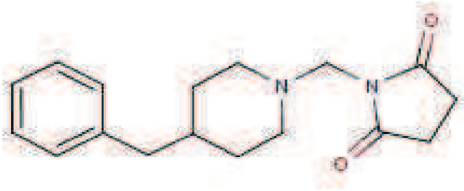 .	O=C1CCC(=O)N1CN1CCC(CC2=CC=CC=C2)CC1	Chembridge (Hit2Lead)	5185654	>90%	<20	0
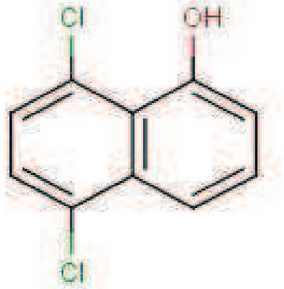 .	OC1=CC=CC2=C1C(Cl)=CC=C2Cl	Chembridge (Hit2Lead)	5211912	>90%	20-70	0

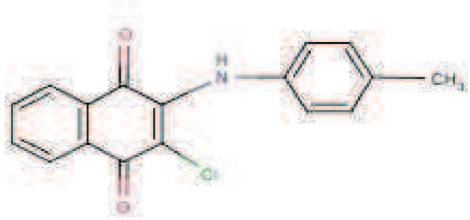	CC1=CC=C(NC2=C(Cl)C(=O)C3=C(C=CC=C3)C2=O)C=C1	Chembridge (Hit2Lead)	5212518	>90%	<20	0
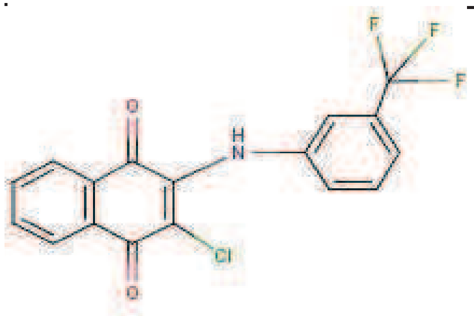	FC(F)(F)C1=CC=CC(NC2=C(Cl)C(=O)C3=C(C=CC=C3)C2=O)=C1	Chembridge (Hit2Lead)	5212519	>90%	<20	0
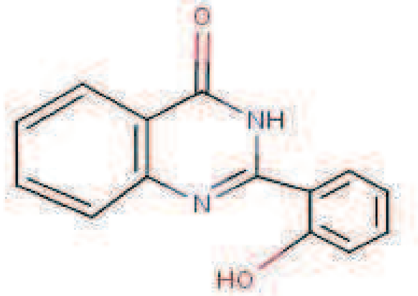	OC1=CC=CC=C1C1=NC2=C(C=CC=C2)C(=O)N1	Chembridge (Hit2Lead)	5213142	>90%	<20	0

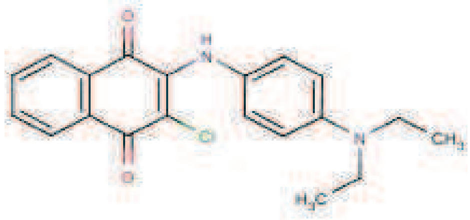		CCN(CC)C1=CC=C(NC2=C(Cl)C(=O)C3=C(C=CC=C3)C2=O)C=C1	Chembridge (Hit2Lead)	5213844	>90%	<20	0
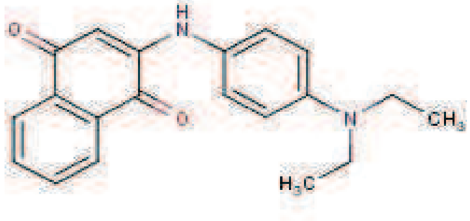		CCN(CC)C1=CC=C(NC2=CC(=O)C3=C(C=CC=C3)C2=O)C=C1	Chembridge (Hit2Lead)	5213850	>90%	<20	0
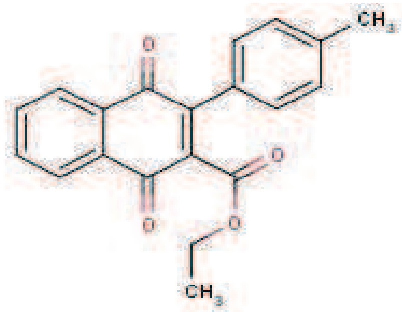		CCOC(=O)C1=C(C(=O)C2=C(C=CC=C2)C1=O)C1=CC=C(C)C=C1	Chembridge (Hit2Lead)	5248599	>90%	70-90	0

. 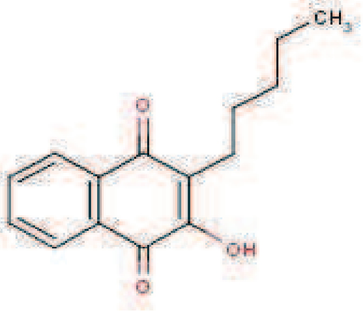		CCCCCC1=C(O)C(=O)C2=C(C=CC=C2)C1=O	Chembridge (Hit2Lead)	5248611	>90%	20-70	0
. 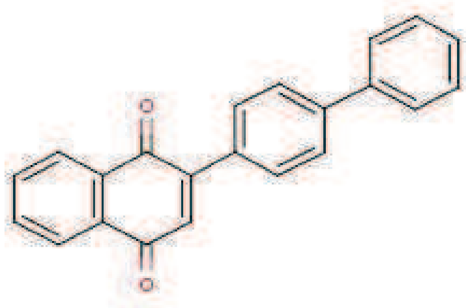		O=C1C=C(C(=O)C2=C1C=CC=C2)C1=CC=C(C=C1)C1=CC=CC=C1	Chembridge (Hit2Lead)	5249293	>90%	<20	0
. 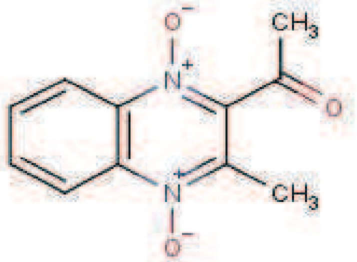		CC(=O)C1=[N+](O)C2=C(C=CC=C2)[N+](O)=C1C	Chembridge (Hit2Lead)	5324085	>90%	<20	0

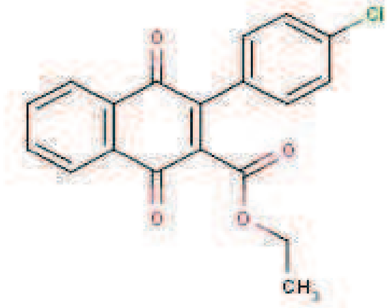 .		CCOC(=O)C1=C(C(=O)C2=C(C=CC=C2)C1=O)C1=CC=C(Cl)C=C1	Chembridge (Hit2Lead)	5325840	>90%	70-90	0
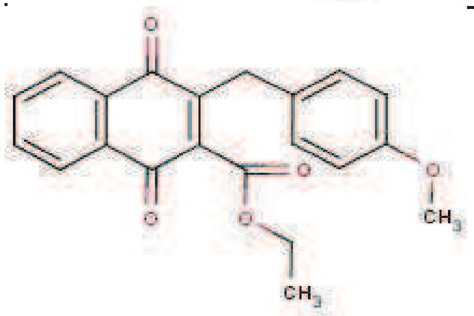 .		CCOC(=O)C1=C(CC2=CC=C(OC)C=C2)C(=O)C2=C(C=CC=C2)C1=O	Chembridge (Hit2Lead)	5325842	>90%	70-90	0
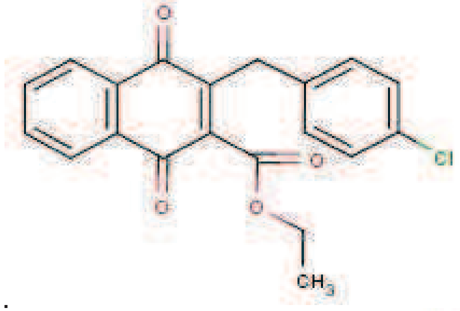 .		CCOC(=O)C1=C(CC2=CC=C(Cl)C=C2)C(=O)C2=C(C=CC=C2)C1=O	Chembridge (Hit2Lead)	5325846	>90%	20-70	0

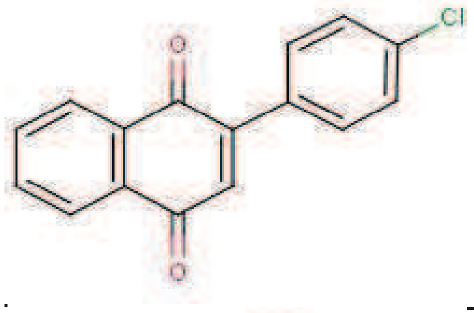 .		ClC1=CC=C(C=C1)C1=CC(=O)C2=C(C=CC=C2)C1=O	Chembridge (Hit2Lead)	5325855	>90%	20-70	0
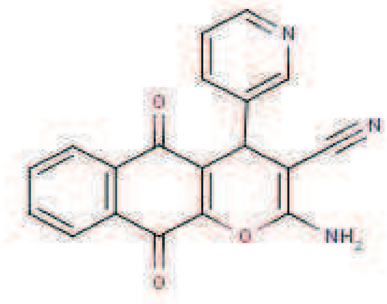 .		NC1=C(C#N)C(C2=CC=CN=C2)C2=C(O1)C(=O)C1=C(C=CC=C1)C2=O	Chembridge (Hit2Lead)	5397895	>90%	20-70	0
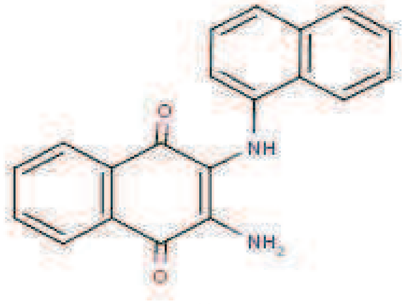 .		NC1=C(NC2CC=CC3=C2C=CC=C3)C(=O)C2=C(C=CC=C2)C1=O	Chembridge (Hit2Lead)	5477554	>90%	<20	0

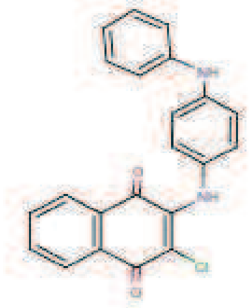 .		ClC1=C(NC2=CC=C(NC3=CC=CC=C3)C=C2)C(=O)C2=C(C=CC=C2)C1=O	Chembridge (Hit2Lead)	5477843	>90%	<20	0
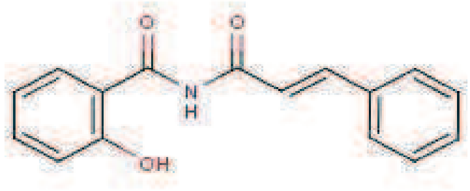 .		OC1=CC=CC=C1C(=O)NC(=O)\C=C\C1=CC=CC=C1	Chembridge (Hit2Lead)	5484914	>90%	<20	0
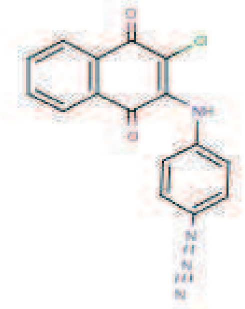 .		ClC1=C(NC2=CC=C(C=C2)N=[N]#N)C(=O)C2=C(C=CC=C2)C1=O	Chembridge (Hit2Lead)	5511359	>90%	20-70	0

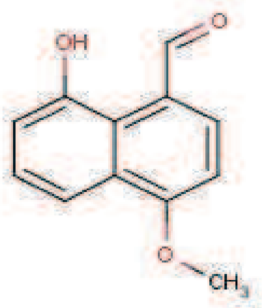 .		COC1=C2C=CC=C(O)C2=C(C=O)C=C1	Chembridge (Hit2Lead)	5565105	>90%	<20	0
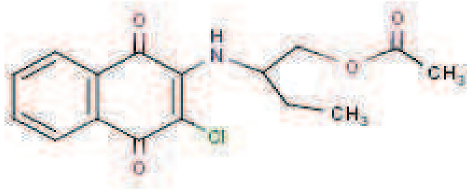 .		CCC(COC(C)=O)NC1=C(Cl)C(=O)C2=C(C=CC=C2)C1=O	Chembridge (Hit2Lead)	5567276	>90%	70-90	0
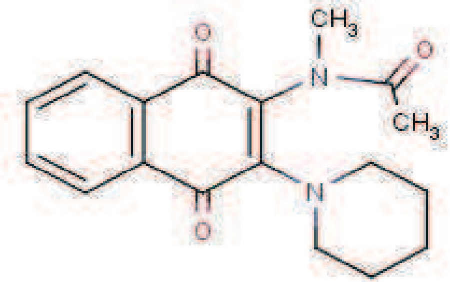 .		CN(C(C)=O)C1=C(N2CCCCC2)C(=O)C2=C(C=CC=C2)C1=O	Chembridge (Hit2Lead)	5604038	>90%	<20	0

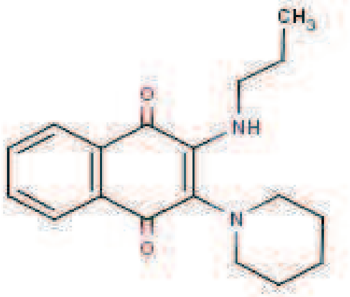 .		CCCNC1=C(N2CCCCC2)C(=O)C2=C(C=CC=C2)C1=O	Chembridge (Hit2Lead)	5605076	>90%	20-70	0
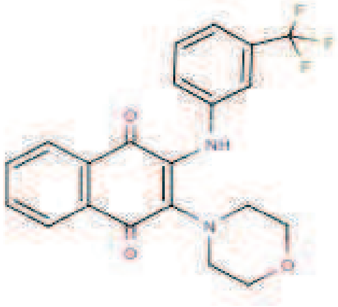 .		CC(C)(C)C1=CC(NC2=C(N3CCOCC3)C(=O)C3=C(C=CC=C3)C2=O)=CC=C1	Chembridge (Hit2Lead)	5609872	>90%	<20	0
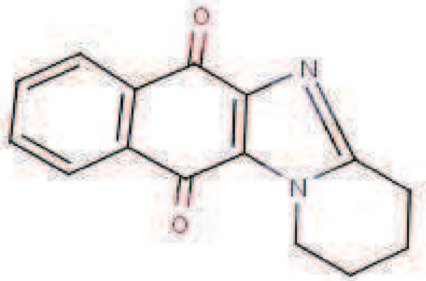 .		O=C1C2=C(N3CCCCC3=N2)C(=O)C2=C1C=CC=C2	Chembridge (Hit2Lead)	5626653	>90%	<20	0

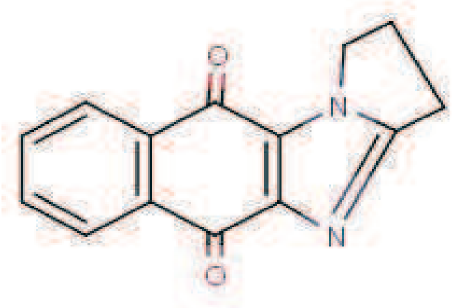		O=C1C2=C(N3CCCC3=N2)C(=O)C2=C1C=CC=C2	Chembridge (Hit2Lead)	5627696	>90%	<20	0
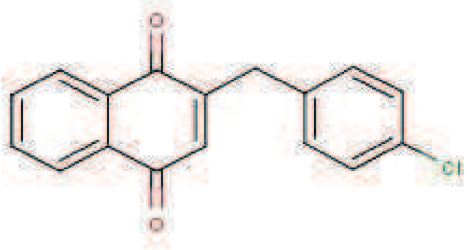		ClC1=CC=C(CC2=CC(=O)C3=C(C=CC=C3)C2=O)C=C1	Chembridge (Hit2Lead)	5706593	>90%	70-90	0
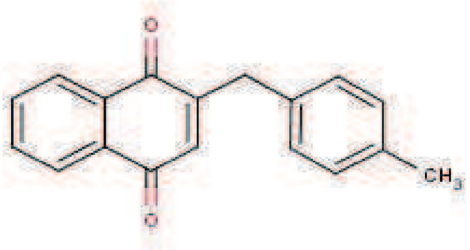		CC1=CC=C(CC2=CC(=O)C3=C(C=CC=C3)C2=O)C=C1	Chembridge (Hit2Lead)	5715755	>90%	90	1

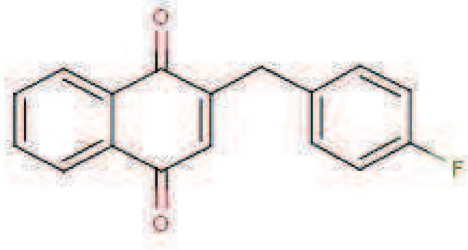 .		FC1=CC=C(CC2=CC(=O)C3=C(C=CC=C3)C2=O)C=C1	Chembridge (Hit2Lead)	5719928	>90%	90	1
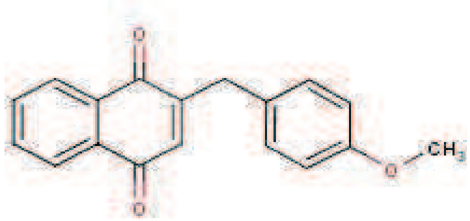 .		COC1=CC=C(CC2=CC(=O)C3=C(C=CC=C3)C2=O)C=C1	Chembridge (Hit2Lead)	5724526	>90%	90	1
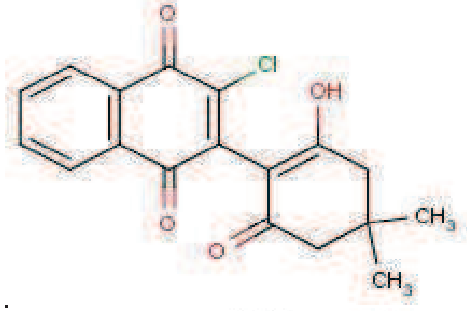 .		CC1(C)CC(O)=C(C(=O)C1)C1=C(Cl)C(=O)C2=C(C=CC=C2)C1=O	Chembridge (Hit2Lead)	5767783	>90%	70-90	0

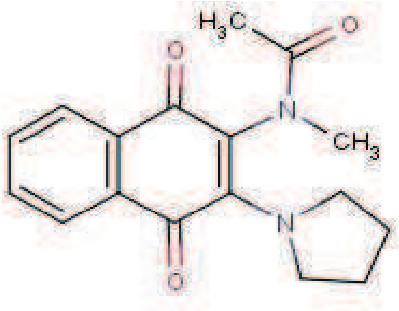 .		CN(C(C)=O)C1=C(N2CCCC2)C(=O)C2=C(C=CC=C2)C1=O	Chembridge (Hit2Lead)	5812253	>90%	<20	0
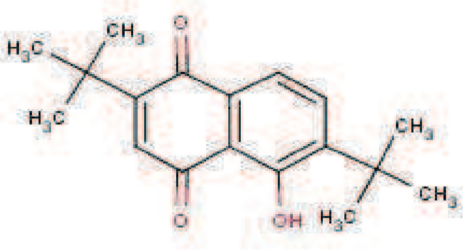 .		CC(C)(C)C1=CC(=O)C2=C(C=CC(=C2O)C(C)(C)C)C1=O	Chembridge (Hit2Lead)	5914723	>90%	<20	0
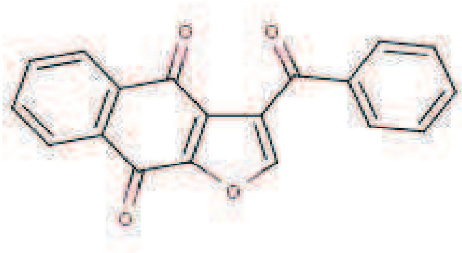 .		O=C(C1=COC2=C1C(=O)C1=C(C=CC=C1)C2=O)C1=CC=CC=C1	Chembridge (Hit2Lead)	6045925	>90%	90	1

. 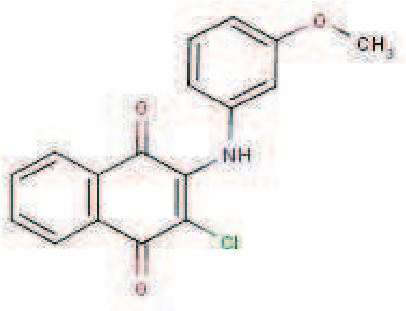		COC1=CC(NC2=C(Cl)C(=O)C3=C(C=CC=C3)C2=O)=CC=C1	Chembridge (Hit2Lead)	6575259	>90%	20-70	0
. 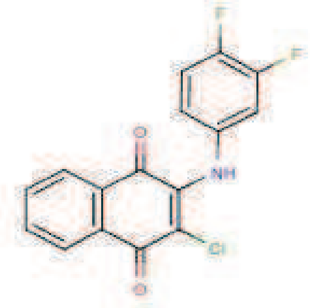		FC1=CC=C(NC2=C(Cl)C(=O)C3=C(C=CC=C3)C2=O)C=C1F	Chembridge (Hit2Lead)	6575573	>90%	<20	0
. 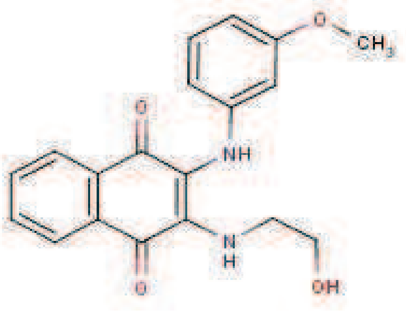		COC1=CC(NC2=C(NCCO)C(=O)C3=C(C=CC=C3)C2=O)=CC=C1	Chembridge (Hit2Lead)	6575733	>90%	<20	0

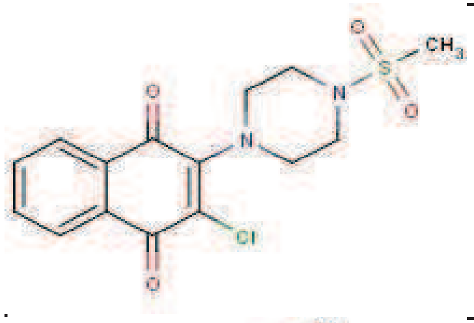 .		CS(=O)(=O)N1CCN(CC1)C1=C(Cl)C(=O)C2=C(C=CC=C2)C1=O	Chembridge (Hit2Lead)	6787496	>90%	90	1
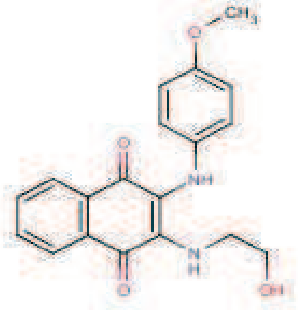 .		COC1=CC=C(CC2=C(NCCO)C(=O)C3=C(C=CC=C3)C2=O)C=C1	Chembridge (Hit2Lead)	6798968	>90%	<20	0
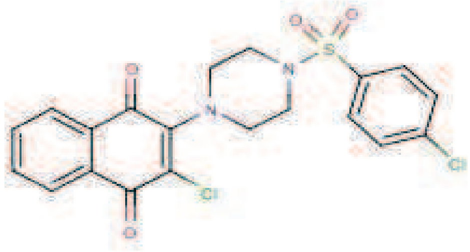 .		ClC1=CC=C(C=C1)S(=O)(=O)N1CCN(CC1)C1=C(Cl)C(=O)C2=C(C=CC=C2)C1=O	Chembridge (Hit2Lead)	6804053	>90%	<20	0

. 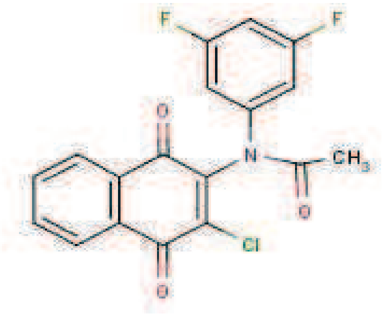		CC(=O)N(C1=CC(F)=CC(F)=C1)C1=C(Cl)C(=O)C2=C(C=CC=C2)C1=O	Chembridge (Hit2Lead)	6845486	>90%	20-70	0
. 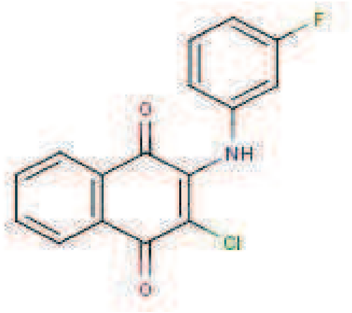		FC1=CC(NC2=C(Cl)C(=O)C3=C(C=CC=C3)C2=O)=CC=C1	Chembridge (Hit2Lead)	6848327	>90%	20-70	0
. 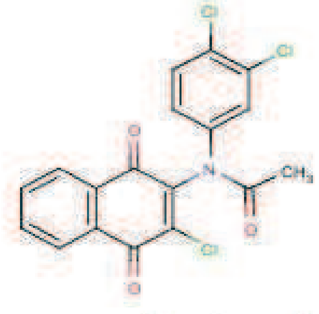		CC(=O)N(C1=CC=C(Cl)C(Cl)=C1)C1=C(Cl)C(=O)C2=C(C=CC=C2)C1=O	Chembridge (Hit2Lead)	6854599	>90%	90	1

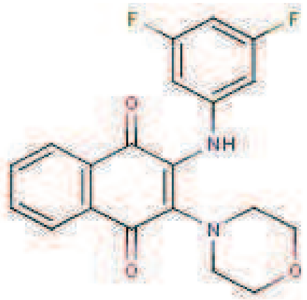 .		FC1=CC(NC2=C(N3CCOCC3)C(=O)C3=C(C=CC=C3)C2=O)=CC(F)=C1	Chembridge (Hit2Lead)	6854963	>90%	<20	0
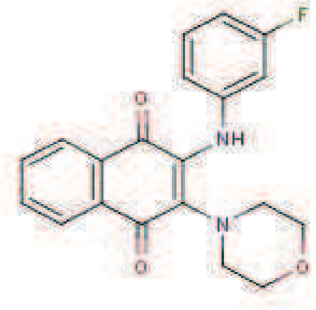 .		FC1=CC(NC2=C(N3CCOCC3)C(=O)C3=C(C=CC=C3)C2=O)=CC=C1	Chembridge (Hit2Lead)	6856548	>90%	<20	0
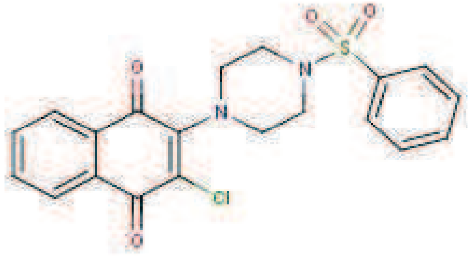 .		ClC1=C(N2CCN(CC2)S(=O)(=O)C2=CC=CC=C2)C(=O)C2=C(C=CC=C2)C1=O	Chembridge (Hit2Lead)	6868264	>90%	20-70	0

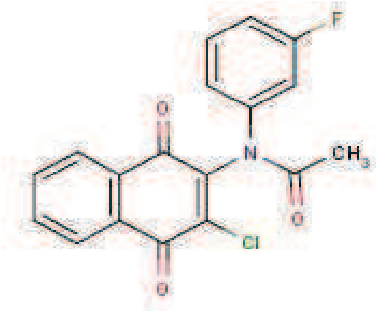		CC(=O)N(C1=CC=CC(F)=C1)C1=C(Cl)C(=O)C2=C(C=CC=C2)C1=O	Chembridge (Hit2Lead)	6868429	>90%	70-90	0
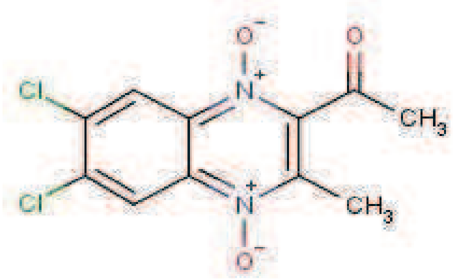		CC(=O)C1=C(C)[N+](O)=C2C=C(Cl)C(Cl)=CC2=[N+]1O	Chembridge (Hit2Lead)	6981662	>90%	<20	0
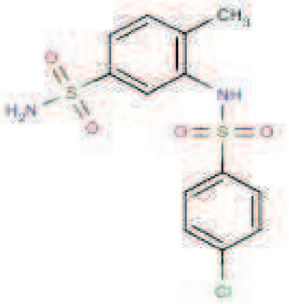		CC1=CC=C(C=C1NS(=O)(=O)C1=CC=C(Cl)C=C1)S(N)(=O)=O	Chembridge (Hit2Lead)	6996892	>90%	<20	0

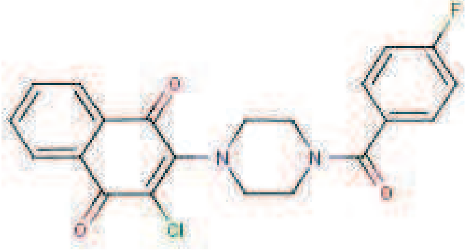		FC1=CC=C(C=C1)C(=O)N1CCN(CC1)C1=C(Cl)C(=O)C2=C(C=CC=C2)C1=O	Chembridge (Hit2Lead)	7044462	>90%	90	1
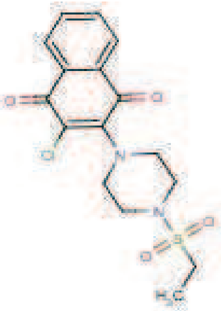		CCS(=O)(=O)N1CCN(CC1)C1=C(Cl)C(=O)C2=C(C=CC=C2)C1=O	Chembridge (Hit2Lead)	7070376	>90%	90	1
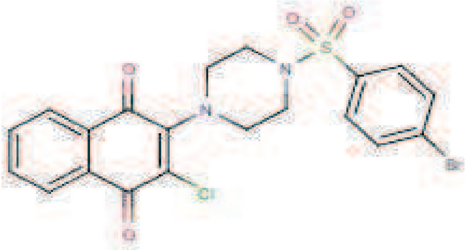		ClC1=C(N2CCN(CC2)S(=O)(=O)C2=CC=C(Br)C=C2)C(=O)C2=C(C=CC=C2)C1=O	Chembridge (Hit2Lead)	7085688	>90%	<20	0

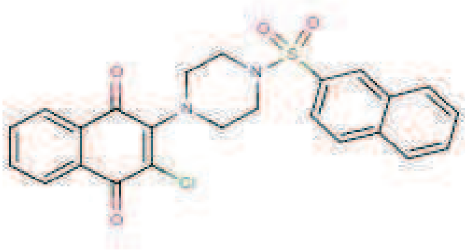 .		ClC1=C(N2CCN(CC2)S(=O)(=O)C2=CC=C3C=CC=CC3=C2)C(=O)C2=C(C=CC=C2)C1=O	Chembridge (Hit2Lead)	7276470	>90%	<20	0
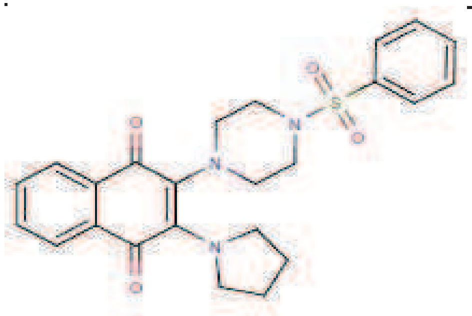 .		O=C1C2=C(C=CC=C2)C(=O)C(N2CCN(CC2)S(=O)(=O)C2=CC=CC=C2)=C1N1CCCC1	Chembridge (Hit2Lead)	7360177	>90%	<20	0
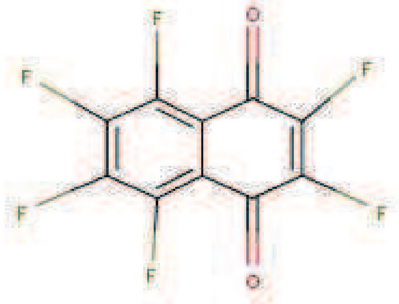 .		FC1=C(F)C2=C(C(F)=C1F)C(=O)C(F)=C(F)C2=O	Chembridge (Hit2Lead)	7404818	>90%	70-90	0

. 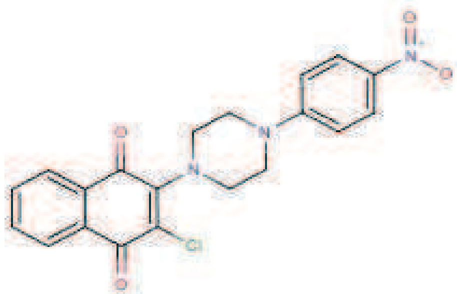		ON(=O)C1=CC=C(C=C1)N1CCN(CC1)C1=C(Cl)C(=O)C2=C(C=CC=C2)C1=O	Chembridge (Hit2Lead)	7617556	>90%	20-70	0
. 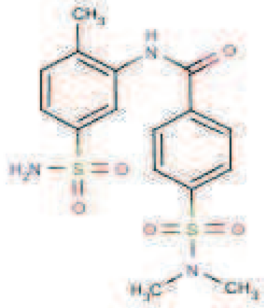		CN(C)S(=O)(=O)C1=CC=C(C=C1)C(=O)NC1=C(C)C=CC(=C1)S(N)(=O)=O	Chembridge (Hit2Lead)	7631270	>90%	<20	0
. 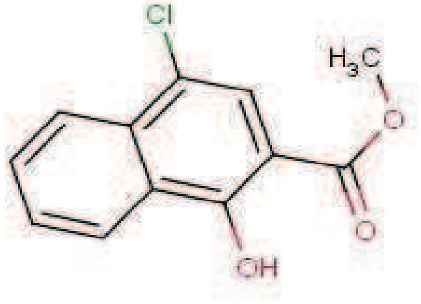		O.COC(=C)C1=C(O)C2=C(C=CC=C2)C(Cl)=C1	Chembridge (Hit2Lead)	7812749	>90%	<20	0

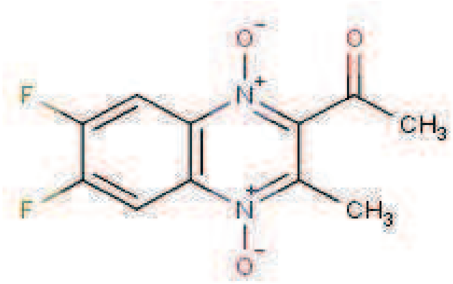 .		CC(=O)C1=[N+](O)C2=C(C=C(F)C(F)=C2)[N+](O)=C1C	Chembridge (Hit2Lead)	8888844	>90%	<20	0
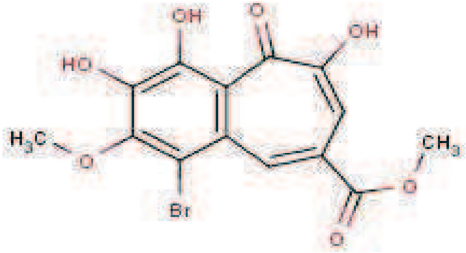 .		COC(=O)C1CC(O)C(=O)C2=C(C1)C(Br)=C(OC)C(O)=C2O	Chembridge (Hit2Lead)	8895247	>90%	20-70	0
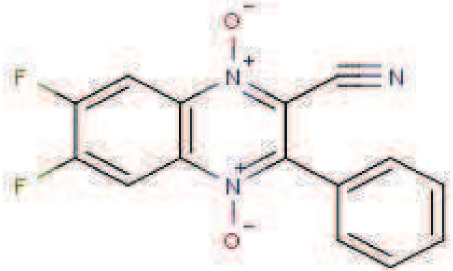 .		O[N+]1=C(C#N)C(C2=CC=CC=C2)=[N+](O)C2=C1C=C(F)C(F)=C2	Chembridge (Hit2Lead)	8895724	>90%	20-70	0

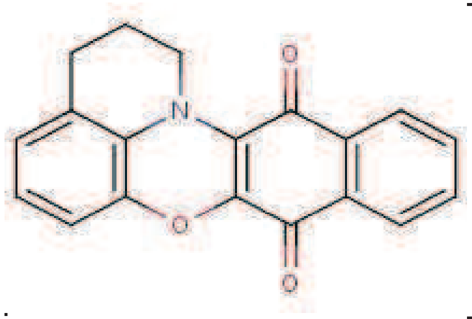	O=C1C2=C(N3CCCC4=CC=CC(O2)=C34)C(=O)C2=C1C=CC=C2	Chembridge (Hit2Lead)	9001794	>90%	20-70	0
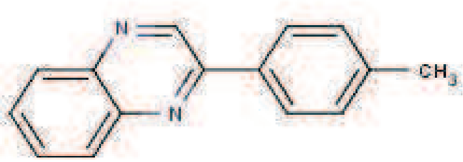	CC1=CC=C(C=C1)C1=NC2=CC=CC=C2N=C1	Chembridge (Hit2Lead)	9032631	>90%	<20	0
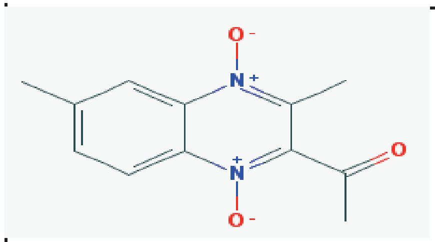	CC(=O)C1=[N+](O)C2	Vitas-M Laboratory, Ltd.	lPort-000-713-5	>90%	<20	0
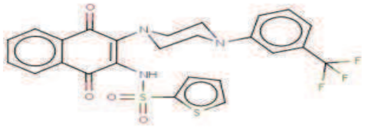	FC(F)(F)C1=CC(=CC=C	InterBioScreen	MolPort-000-635-294	92-95%	<20	0

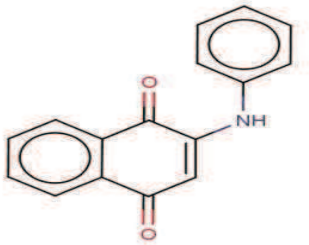	O=C1C=C(NC2=CC=C	InterBioScreen	MolPort-000-775-383	92-95%	20-70	0
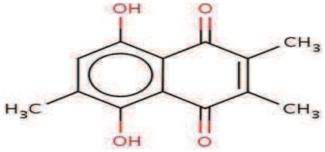	CC1=C(O)C2=C(C(O)=	InterBioScreen	MolPort-002-525-817	92-95%	20-70	0
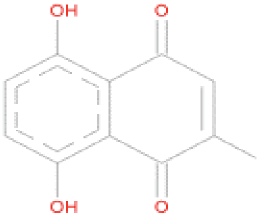	CC1=CC(=O)c2c(O)cc	InterBioScreen	MolPort-002-525-117	92-95%	90	1
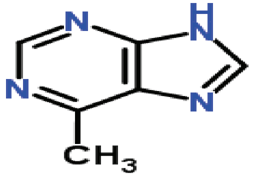	CC1=NC=NC2=C1N=C	Sigma	sigma M6502	>99%	<20	0

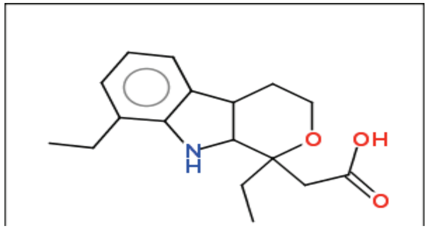	CCC1=CC2=C(C=C1)C	Sigma	Sigma E0516	>98%	<20	0
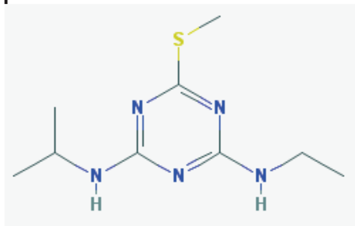	CCNC1=NC(NC(C)C)=	Sigma	Sigma 45321		<20	0
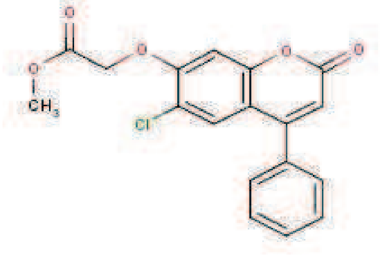	COC(=O)COC1=CC2=(	Chembridge (Hit2Lead)	5618162	>90%	<20	0
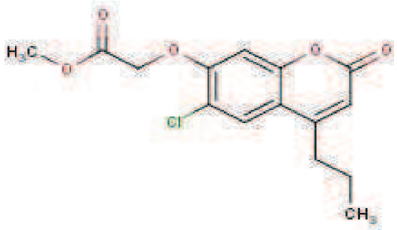	CCCC1=CC(=O)OC2=(	Chembridge (Hit2Lead)	5753116	>90%	<20	0

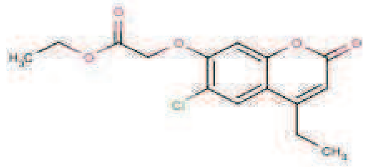	CCOC(=O)COC1=CC2	Chembridge (Hit2Lead)	6653343	>90%	<20	0
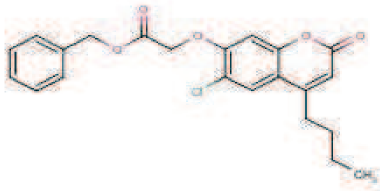	CCCCC1=CC(=O)OC2=	Chembridge (Hit2Lead)	6661898	>90%	<20	0
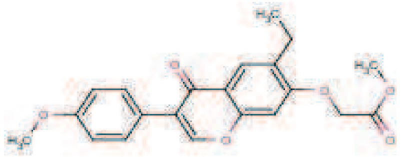	CCC1=C(OCC(=O)OC)	Chembridge (Hit2Lead)	6939246	>90%	<20	0
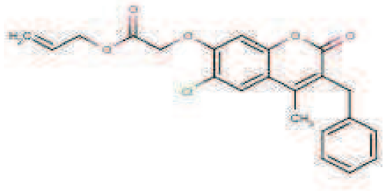	CCC1=C(C)C2=C(OC1	Chembridge (Hit2Lead)	7105644	>90%	<20	0

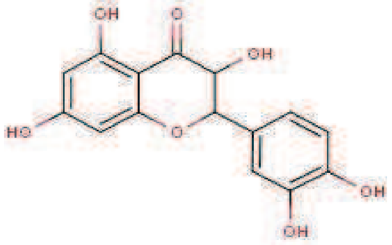	OC1C(OC2=C(C(O)=C	Chembridge (Hit2Lead)	6101281	>90%	<20	0
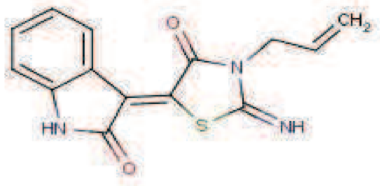	C=CCN1C(=N)S\C(C1	Chembridge (Hit2Lead)	5679728	>90%	<20	0
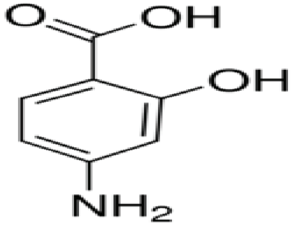	NC1=CC(O)=C(C=C1)	Sigma	Sigma A79604		<20	0
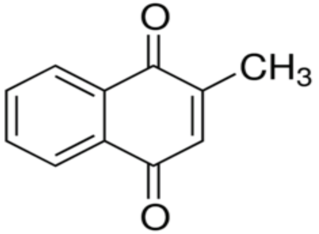	CC1=CC(=O)C2=C(C=	Sigma	Sigma M5625		90	1

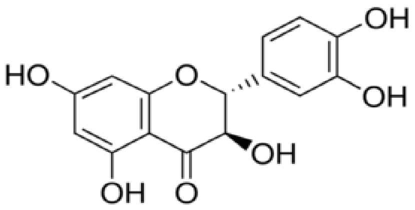	OC1C(OC2=C(C(O)=C	Sigma	Sigma 78666	>85%	<20	0
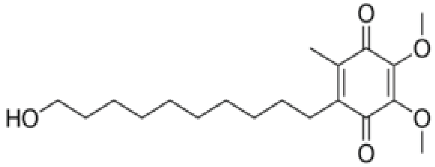	COC1C(OC)C(=O)C(C(	Sigma	Sigma I5659	>98%	90	1

Supplementary Table 7. Bayesian scores and experimental results for NQs in a test set. a = Bayesian score, higher values are better, b = scores greater than 3.399 = active, c = closest distance to compounds in training set, d = actual measured data. Note that higher Bayesian scores based on the features in each molecule scored as active or inactive predict more reliably inhibition.

Structure	Code	Bayesian Score [a]	Prediction for activity [b]	Closest distance[c]	% of ThyX inhibition at 100 µM [d]
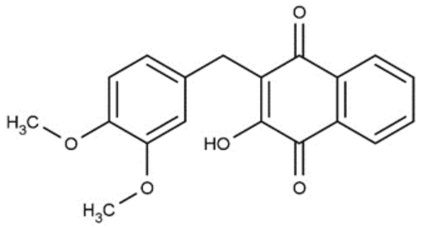	010K	5.23	TRUE	0.56	42
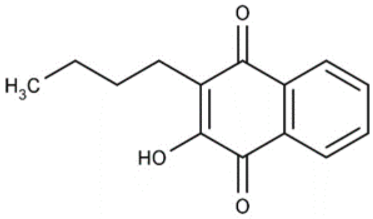	010I	1.88	FALSE	0.16	58
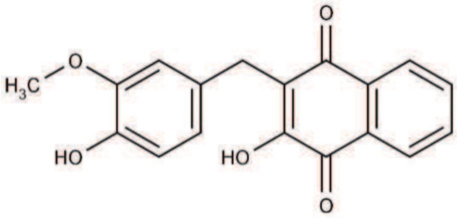	010H	3.89	TRUE	0.62	41
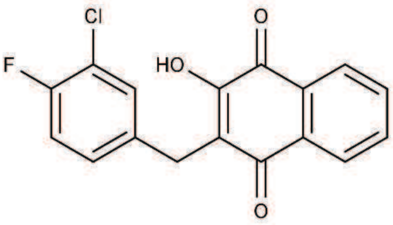	010F	8.6	TRUE	0.44	73

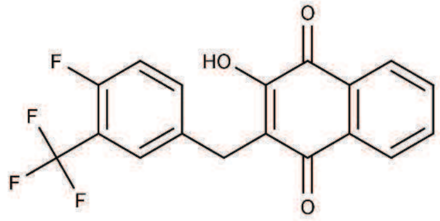	010E	7.81	TRUE	0.44	32
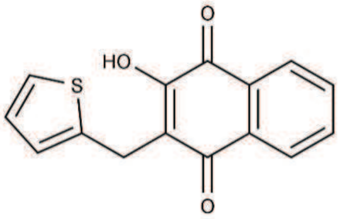	010D	3.68	TRUE	0.67	42
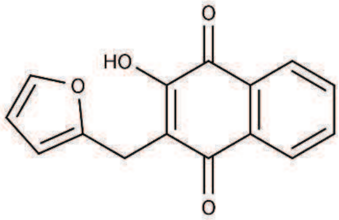	010C	2.72	FALSE	0.62	60
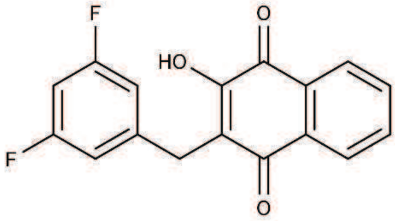	010B	6.18	TRUE	0.44	68
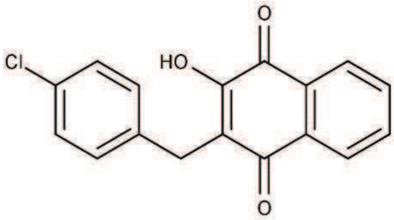	010A	10.13	TRUE	0.45	64
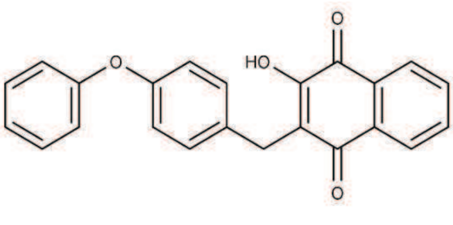	007G	5.73	TRUE	0.59	89

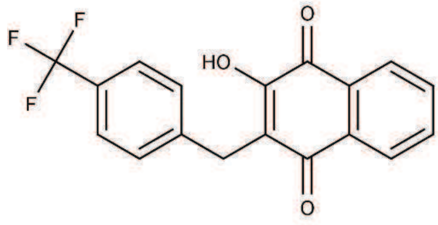	007F	7.37	TRUE	0.45	74
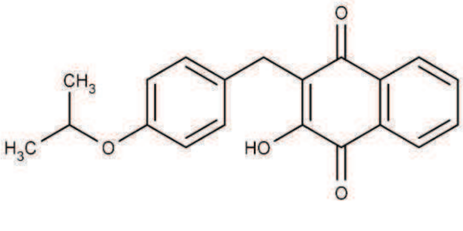	007D	8.28	TRUE	0.50	71
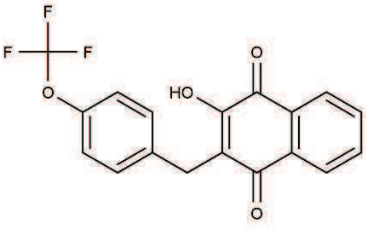	007C	8.23	TRUE	0.57	78
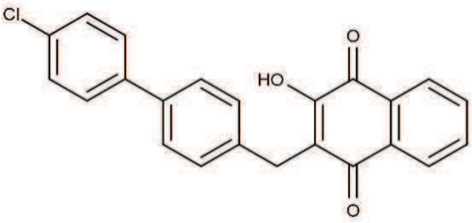	007B	6.48	TRUE	0.66	21

Supplementary Table 8. Strains used to test the whole-cell activity of the NQs in the test set for the Bayesian model.

Strain	MIC (µM)		Resistance
	RMP	INH	
**H37Rv**	<0.0.30	<0.91	none
**MT 182**	1.21	<0.91	RMP
**TC 12561**	4.86	14.58	RMP+ INH
**TH 12526**	<0.30	<0.91	none
**TH12768**	<0.30	3.64	INH

RMP- rifampicin, INH- isoniazid, RMP MIC is 0.5 µg/ml and INH MIC is 0.25 µg/ml[21]

Supplementary Table 9. Antibacterial activities of the fourteen NQs in the test set.

Compound	MIC (μM)				
	H37Rv	MT 182	TC 12561	TH 12526	TH 12768
**010A**	52.3	104.6	52.3	52.3	104.6
**007B**	20.8	83.4	83.4	41.7	41.7
**010B**	104.1	104.1	104.1	104.1	52.1
**007C**	89.7	44.9	44.9	44.9	44.9
**010C**	176.5	176.5	176.5	176.5	176.5
**007D**	96.9	96.9	96.9	96.9	96.9
**010D**	231.2	462.4	57.8	231.2	231.2
**010E**	22.3	22.3	22.3	22.3	22.3
**007F**	94.1	94.1	47.0	47.0	94.1
**010F**	197.3	98.7	49.3	98.7	197.3
**007G**	175.5	87.8	87.8	87.8	175.5
**010H**	201.4	100.7	201.4	201.4	201.4
**010I**	135.7	135.7	271.4	67.8	135.7
**010K**	192.7	385.4	192.7	48.2	192.7
